# Mechanism-aware personalized learning intervention for short- and long-term academic outcome modeling

**DOI:** 10.3389/fpsyg.2026.1831489

**Published:** 2026-05-14

**Authors:** Lei Zhang

**Affiliations:** Jinhua University of Vocational Technology, Jinhua, Zhejiang, China

**Keywords:** academic achievement modeling, affective support, AI-driven personalized learning, learner-state representation, long-term academic outcomes, mechanism-aware intervention, self-regulated learning

## Abstract

**Introduction:**

Improving academic achievement while also modeling students' longer-horizon academic development has become an important goal in educational psychology and AI-supported learning research. However, adaptive learning, learner modeling, feedback generation, and academic prediction are often studied separately, and the learner conditions used to guide intervention are not always defined with sufficient theoretical and methodological transparency.

**Methods:**

This study proposes **MAPLe-I** (**M**echanism-**A**ware **P**ersonalized **Le**arning **I**ntervention), a unified framework that integrates learner-state encoding, theory-informed latent proxy estimation, personalized intervention policy learning, and dual-horizon academic outcome modeling. Learner state is encoded from graph-structured knowledge relations and temporal behavioral sequences, and the model estimates three intervention-relevant latent proxies: engagement-related state, self-regulation-related readiness, and affective support need. Because the public datasets do not include observed intervention decisions, policy supervision was implemented using rule-derived pedagogical pseudo-labels. The framework was evaluated offline on ASSISTments 2012–2013 with Affect, EdNet-KT1, and OULAD against non-personalized digital intervention, traditional adaptive learning, AI-based feedback, and learner-modeling or academic-prediction baselines.

**Results:**

MAPLe-I achieved stronger comparative benchmark performance across multiple settings. On ASSISTments, it obtained an AUC of 0.824 and an Intervention Alignment Rate of 0.785. On EdNet-KT1, it obtained an AUC of 0.812. On OULAD, it achieved a Macro-F1 of 0.772 and reduced RMSE to 0.315. Ablation and sensitivity analyses further supported the contribution of the temporal encoder, mechanism head, intervention policy head, and dual-horizon optimization strategy.

**Discussion:**

The findings suggest that integrating learner-state diagnosis, theory-informed latent proxies, and intervention-policy modeling can improve offline predictive and rule-alignment performance in benchmark settings. However, the mechanism variables are computational proxies, the intervention labels are rule-derived rather than observed, and the results do not establish causal effects or authentic classroom impact. MAPLe-I should therefore be interpreted as a transparent benchmark framework for mechanism-aware personalized learning rather than as a validated real-world intervention system.

## 1 Introduction

Improving students' academic achievement while simultaneously supporting their long-term developmental potential has become a central concern in contemporary educational research. In increasingly digital and data-rich learning environments, students are exposed to abundant instructional resources, automated practice systems, and intelligent tutoring platforms. However, access to digital learning opportunities does not automatically translate into improved academic outcomes. A recurring challenge in both educational psychology and educational technology is that learners differ substantially in prior knowledge, engagement patterns, self-regulatory capacity, affective conditions, and responsiveness to instructional support (Fredricks et al., 2004; Zimmerman, 1989, 2002; Pintrich, 2004; Pekrun, 2006). As a result, uniform instructional treatment often fails to meet the needs of heterogeneous learners, particularly those whose current academic performance does not fully reflect their learning potential (Dockterman, 2018; Dumont and Ready, 2023; Brummelman et al., 2024).

Against this backdrop, AI-driven personalized learning has attracted growing scholarly attention as a promising means of improving learning efficiency, tailoring instructional support, and identifying students at risk of underachievement (Wang et al., 2024a; Holmes and Tuomi, 2022; Hariyanto et al., 2025; Fortuna et al., 2025). Recent advances in educational data mining, learning analytics, knowledge tracing, adaptive platforms, and generative AI have made it possible to model learner behavior at unprecedented scale and granularity (Shen et al., 2024; Tan et al., 2025; Xia et al., 2026; Reich, 2015). These developments have encouraged researchers to design systems that adapt content difficulty, generate feedback, predict academic performance, and recommend learning resources based on learner data (Wang et al., 2024b; Contrino et al., 2024; Na Nongkhai et al., 2026). In principle, such approaches offer considerable promise for supporting more individualized and responsive forms of education (VanLehn, 2011; Ma et al., 2014; Koedinger et al., 2012; Chirikov et al., 2020; Kizilcec et al., 2020).

Recent pathway-oriented work has further emphasized AI-driven personalized learning pathways and adaptive content delivery systems as practical routes for tailoring support to different learner profiles (Palaniappan et al., 2025). This line of work is directly relevant to the present study because it reinforces the need to connect learner diagnosis with actionable personalized support rather than treating personalization as static content matching alone.

Nevertheless, an important conceptual and methodological gap remains. Many current AI-based educational systems emphasize one specific function, such as adaptive content delivery, learner-state prediction, performance forecasting, or feedback generation (Contrino et al., 2024; Shen et al., 2024; Na Nongkhai et al., 2026; Wang and Luo, 2024). Although these functions are valuable, they do not fully explain *why* personalized intervention improves academic achievement, nor do they adequately capture the mechanisms through which different learners respond to support. In other words, much of the existing literature has focused on whether AI systems can optimize learning outputs, but less attention has been paid to the psychologically meaningful processes that mediate the relationship between AI-driven intervention and academic improvement (Lan and Zhou, 2025; Xia et al., 2026; Zhang et al., 2025). This limitation is particularly salient from an educational psychology perspective, where learning outcomes are understood not merely as products of content exposure, but as emergent consequences of complex interactions among cognition, motivation, self-regulation, affect, and contextual support (Fredricks et al., 2004; Pekrun, 2006; Panadero, 2017).

This issue is especially important when considering the broader educational goal of *unlocking students' potential*. Academic achievement is not simply a reflection of current performance; it is also shaped by whether learners receive support that is appropriately aligned with their readiness, engagement condition, and developmental needs (Dumont and Ready, 2023; Brummelman et al., 2024; Cullen and Oppenheimer, 2024). Students who appear similar in achievement data may differ substantially in persistence, emotional support need, or capacity to benefit from challenge and scaffolding (Fredricks et al., 2004; Zimmerman, 2002; Pekrun, 2006). Consequently, systems that rely only on observable performance or static adaptation rules may fail to identify the deeper mechanisms that determine intervention effectiveness. From this perspective, AI-driven personalized learning should not be treated merely as an optimization problem, but as a mechanism-sensitive instructional process.

The present study was motivated by this theoretical and practical need. Specifically, this research asks whether AI-driven personalized learning interventions can improve students' academic achievement more effectively when they explicitly incorporate intervention-relevant learner mechanisms. Rather than treating prediction, adaptation, and feedback as separate tasks, this study proposes that effective personalized intervention requires an integrated framework that connects learner-state diagnosis, mechanism estimation, intervention planning, and short-term as well as long-term academic outcome optimization.

Accordingly, the present study addresses the following core research problem: *How can AI-driven personalized learning interventions improve academic achievement while simultaneously supporting students' developmental potential through mechanism-aware support?* This overarching question can be further decomposed into three more specific questions. First, can personalized intervention outperform non-personalized or traditional adaptive instructional approaches? Second, can a mechanism-aware intervention framework outperform feedback-centered or prediction-centered AI baselines? Third, can such a framework improve not only immediate learning performance but also broader academic development over time? These questions directly connect the technical design of the proposed model with the educational psychological concern of understanding how instructional effectiveness emerges.

The purpose of this study is therefore threefold. First, it aims to develop a theoretically grounded AI framework for personalized learning intervention that goes beyond isolated functions such as prediction or feedback generation. Second, it seeks to model latent intervention-relevant learner mechanisms, specifically engagement-related state, self-regulation-related readiness, and affective support need, as intermediate factors linking learner data to instructional decision-making. Third, it aims to empirically evaluate whether such a mechanism-aware framework can yield stronger benchmark performance in both short-term learning prediction and long-term academic outcome modeling across multiple public educational datasets.

Achieving these goals involves several non-trivial research challenges. The first challenge lies in learner heterogeneity. Students differ not only in prior knowledge, but also in how they engage with learning tasks, how persistently they regulate their learning behavior, and how they respond to frustration, confusion, or support (Fredricks et al., 2004; Zimmerman, 1989; Panadero, 2017; Pekrun, 2006). Capturing these differences in a computationally meaningful form is difficult, especially when direct psychological measurements are unavailable. The second challenge concerns the fragmented nature of existing methods. Adaptive learning systems often focus on content sequencing, feedback models focus on response generation, and prediction models focus on future outcomes, but these components are rarely unified into a single intervention-oriented framework (Contrino et al., 2024; Na Nongkhai et al., 2026; Wang et al., 2025; Ren and Yu, 2024). The third challenge lies in aligning short-term optimization with long-term development. A system may improve next-step correctness without necessarily contributing to sustained academic improvement or potential realization (Ren and Yu, 2024; Wang and Luo, 2024; Dumont and Ready, 2023). The fourth challenge concerns interpretability. In educational and psychological research, it is not sufficient to report that a model performs well; it is also important to explain what learner conditions the model is responding to and why its intervention strategy is educationally meaningful (Wang and Luo, 2024; Zhang et al., 2025; Koedinger et al., 2012).

These challenges motivate the central idea of the present study: personalized learning interventions should be both *AI-driven* and *mechanism-aware*. The main departure point of the proposed approach is that educationally effective intervention requires more than accurate prediction or sophisticated feedback. Instead, effective intervention should be based on a structured representation of learner state, an estimation of latent learner mechanisms relevant to intervention, and a policy that transforms those estimates into adaptive pedagogical actions. In this way, the model does not merely identify whether a student is likely to struggle or succeed; it also attempts to determine what type of support is most appropriate given the learner's current condition.

Based on this idea, this study proposes **MAPLe-I** (**M**echanism-**A**ware **P**ersonalized **Le**arning **I**ntervention), a unified framework for AI-driven personalized learning intervention. MAPLe-I integrates four core components: a learner-state encoder, a mechanism estimation head, an intervention policy head, and a dual outcome head. First, the learner-state encoder combines graph-structured knowledge information with temporal behavioral interaction data to generate a comprehensive representation of the learner's evolving learning state. Second, the mechanism estimation head infers latent intervention-relevant mechanisms, including engagement-related state, self-regulation-related readiness, and affective support need. Third, the intervention policy head generates personalized support actions, such as feedback selection, hint allocation, difficulty adjustment, remedial support, and pacing control. Fourth, the dual outcome head jointly models short-term learning performance and long-term academic development, allowing the framework to optimize both immediate and cumulative educational benefit.

The logic of MAPLe-I is therefore fundamentally different from existing baselines that focus on only one aspect of intelligent educational support. Compared with non-personalized digital instruction, the proposed framework explicitly adapts support to learner differences. Compared with traditional adaptive learning, it is not limited to rule-based path adjustment (Contrino et al., 2024). Compared with adaptive, generative-AI, or hybrid feedback baselines, it treats feedback as only one component of a broader intervention strategy (Na Nongkhai et al., 2026). Compared with knowledge tracing and academic prediction baselines, it moves from diagnosis to action by converting learner-state information into personalized support decisions (Wang et al., 2025; Ren and Yu, 2024; Wang and Luo, 2024). This integrated architecture is intended to offer a more theoretically meaningful and methodologically transparent account of how AI-driven personalized intervention may be modeled in relation to academic achievement.

To evaluate the proposed framework, this study employs a multi-dataset experimental design using publicly available educational datasets with complementary characteristics. ASSISTments 2012–2013 with Affect is used as the primary dataset because it provides fine-grained interaction logs and affect-related signals, which are suitable for mechanism-aware modeling (Heffernan and Heffernan, 2014). EdNet-KT1 is used to test scalability and cross-dataset generalization in a large-scale sequential learning environment (Choi et al., 2020b). OULAD is used to evaluate long-term academic outcomes at the course level (Kuzilek et al., 2017). Across these datasets, MAPLe-I is compared against a carefully selected set of baselines, including non-personalized digital intervention, traditional adaptive learning, adaptive feedback, generative-AI feedback, hybrid feedback, GKTP, LASA, and XGB-SHAP. This baseline design allows the study to test whether the proposed framework offers advantages over conventional, feedback-centered, and prediction-centered approaches.

The empirical results reported in the later chapters suggest that MAPLe-I consistently outperforms the selected baselines across short-term learning performance, long-term academic outcome prediction, intervention alignment, and potential-improvement estimation. These findings indicate that AI-driven personalized learning becomes more effective when it is not reduced to adaptive delivery, feedback generation, or risk prediction alone, but is instead designed as a mechanism-aware intervention process. From a theoretical standpoint, this supports the view that academic achievement improvement is best understood as an outcome of the alignment between learner condition and pedagogical response. From a practical standpoint, it suggests that future educational AI systems should integrate diagnosis, mechanism estimation, and intervention planning rather than treating them as isolated tasks.

This study makes four main contributions. First, it reconceptualizes AI-driven personalized learning as a mechanism-aware intervention problem rather than a narrowly predictive or feedback-generation task. Second, it proposes MAPLe-I, a unified framework that integrates learner-state encoding, latent mechanism estimation, personalized intervention policy learning, and dual-horizon academic outcome modeling. Third, it introduces a psychologically informed intervention design by explicitly modeling engagement-related state, self-regulation-related readiness, and affective support need as latent intervention-relevant mechanisms. Fourth, it provides a multi-dataset empirical evaluation across ASSISTments, EdNet-KT1, and OULAD, demonstrating stronger comparative benchmark performance of the proposed framework across short-term learning prediction, long-term academic outcome modeling, and potential-improvement estimation.

The remainder of this paper is organized as follows. Section 2 reviews the relevant literature on AI-driven personalized learning, adaptive intervention, learner modeling, and educational psychological mechanisms. Section 3 presents the proposed MAPLe-I framework in detail. Section 4 reports the experimental design and empirical results. Section 5 discusses the findings from the perspective of educational psychology and AI-driven intervention. Section 6 concludes the study, outlines limitations, and suggests directions for future research.

## 2 Related work

This chapter reviews prior research relevant to the present study from two interrelated perspectives: the research background and current state of AI-driven personalized learning, and the major challenges that remain unresolved in the literature. The purpose of this chapter is not merely to summarize existing studies, but to identify the conceptual and methodological gap that motivates the proposed MAPLe-I framework. In line with the objectives of this study, the review focuses on four strands of scholarship: AI-driven personalized learning and adaptive educational systems, learner modeling and academic outcome prediction, AI-mediated feedback and mechanism-relevant support, and the unresolved challenges that constrain current approaches.

### 2.1 Research background and current state

#### 2.1.1 AI-driven personalized learning and adaptive educational systems

Personalized learning has long been regarded as a central goal of education because learners differ substantially in prior knowledge, pace, engagement, and support needs (Dockterman, 2018; Dumont and Ready, 2023; Brummelman et al., 2024). With the rapid development of artificial intelligence in education, researchers have increasingly explored how AI can be used to tailor learning content, feedback, and support strategies to individual learners. Recent reviews suggest that the field has evolved from early rule-based intelligent tutoring systems toward data-driven adaptive platforms, learning analytics, and generative-AI-supported educational applications (Wang et al., 2024a; Holmes and Tuomi, 2022; Zawacki-Richter et al., 2019; Hariyanto et al., 2025; Fortuna et al., 2025; Roll and Wylie, 2016; Anderson et al., 1985). These developments have significantly expanded the technical capacity for personalized learning.

Among these developments, adaptive learning systems represent one of the most established forms of AI-enabled personalization. Such systems typically adjust learning pathways, task sequencing, or resource allocation according to learner performance and interaction history. Prior studies have shown that adaptive learning can improve student performance and learning satisfaction in both online and blended learning environments (Contrino et al., 2024; Tan et al., 2025). However, most adaptive systems still conceptualize personalization primarily as content delivery adjustment, rather than as a mechanism-sensitive intervention process.

#### 2.1.2 Learner modeling and academic outcome prediction

A second major strand of research concerns learner modeling, especially knowledge tracing and academic outcome prediction. Knowledge tracing aims to estimate learners' evolving knowledge states based on interaction histories, thereby enabling systems to predict subsequent performance and personalize instructional support. Recent surveys indicate that knowledge tracing has developed from probabilistic approaches toward deep sequential, graph-based, and Transformer-based models (Shen et al., 2024). These advances have substantially improved the modeling of temporal learning dynamics and concept-level mastery.

Recent transformer-based knowledge tracing models help clarify the current state of the art in this area. Self-Attentive Knowledge Tracing (SAKT), Context-Aware Attentive Knowledge Tracing (AKT), and SAINT have become influential reference models for capturing long-range dependencies in student learning sequences through attention-based architectures (Pandey and Karypis, 2019; Ghosh et al., 2020; Choi et al., 2020a). These models substantially strengthen sequential learner modeling, but they are primarily optimized for next-step performance prediction rather than for producing multi-head pedagogical actions, explicit latent proxy estimates, or dual-horizon academic outcome predictions within a unified intervention framework. In parallel, recent large language model work in education has expanded the landscape of adaptive tutoring, feedback generation, and learning-path personalization (Palaniappan et al., 2025; Shi et al., 2026). However, most LLM-centered studies still foreground conversational assistance or content generation rather than an explicitly benchmarked linkage among learner-state diagnosis, proxy estimation, intervention policy learning, and longer-horizon academic outcome modeling.

In parallel, academic outcome prediction research has developed increasingly sophisticated methods for forecasting student performance, identifying at-risk learners, and supporting educational decision-making. Such methods include graph-based knowledge tracing, long-term performance prediction, and interpretable machine-learning approaches (Wang et al., 2025; Ren and Yu, 2024; Wang and Luo, 2024). Although these models provide valuable predictive information, their primary focus remains diagnostic or predictive rather than intervention-oriented. As a result, many studies can estimate whether a learner is likely to struggle, but do not directly address what form of pedagogical support should be delivered in response.

#### 2.1.3 AI-mediated feedback and psychologically relevant support

A third related line of work focuses on AI-mediated feedback, which has become increasingly prominent with the emergence of natural language processing and generative AI. Recent studies suggest that adaptive feedback, generative feedback, and hybrid feedback paradigms can all improve the immediacy and personalization of learning support, although their effectiveness varies across learning scenarios (Na Nongkhai et al., 2026). At the same time, emerging review studies on generative AI and self-regulated learning indicate that AI has the potential to support learner planning, monitoring, reflection, and adaptive adjustment (Lan and Zhou, 2025; Xia et al., 2026).

In addition, emotionally responsive or affect-sensitive AI has received growing attention in educational psychology and learning sciences. Recent evidence suggests that affect-aware AI systems may help improve learning performance, engagement, and support alignment in certain educational settings (Zhang et al., 2025). However, these lines of work remain relatively fragmented, and they are rarely integrated into a unified mechanism-aware intervention framework.

### 2.2 Major challenges in the existing literature

Although prior research has made substantial progress, several challenges remain unresolved. These challenges directly motivate the present study.

#### 2.2.1 Fragmentation of instructional functions

One major challenge is the fragmentation of instructional functions across the existing literature. Adaptive learning research tends to emphasize pathway adjustment and content sequencing; learner modeling research emphasizes diagnosis and prediction; feedback research emphasizes response generation; and self-regulated learning research emphasizes process support (Contrino et al., 2024; Shen et al., 2024; Na Nongkhai et al., 2026; Lan and Zhou, 2025). Each of these strands contributes something important, but they are often pursued in parallel rather than integrated into a single intervention-oriented framework. Consequently, many existing systems are strong in one function but weak in others.

This fragmentation creates a practical and theoretical limitation. From an educational standpoint, effective personalized intervention requires more than isolated optimization of a single component. Diagnosing the learner, estimating what kind of support is needed, selecting an appropriate intervention, and optimizing learning outcomes are interdependent processes. Yet many current methods still separate these processes, which makes it difficult to explain how intervention effectiveness emerges as a whole.

#### 2.2.2 Weak linkage with educational psychological mechanisms

A second challenge lies in the limited integration of educational psychological mechanisms into computational personalization frameworks. Many AI-based educational systems adapt to observable performance, but do not explicitly model intervention-relevant learner conditions such as engagement, self-regulation-related readiness, or affective support need. Similarly, some recent studies acknowledge the importance of self-regulated learning, motivation, or emotional support, but do not translate these constructs into computational components that can directly guide intervention decisions (Lan and Zhou, 2025; Xia et al., 2026; Zhang et al., 2025).

This limitation matters because learners with similar observable performance may differ substantially in their responsiveness to intervention. For example, one learner may need increased challenge, another may need strategic scaffolding, and another may need affective reassurance. If a system does not model these differences, personalization risks remaining behavior-reactive rather than mechanism-sensitive. Thus, the literature still lacks a sufficiently explicit bridge between educational psychological theory and intervention-oriented AI design.

#### 2.2.3 Tension between short-term optimization and long-term development

A third challenge concerns the tension between short-term optimization and long-term development. Many educational AI models are trained and evaluated using immediate outcomes such as next-response correctness, mastery estimation, or local performance prediction. While such outcomes are important, they do not necessarily capture whether the learner is developing in a broader academic sense. A system can improve short-term accuracy without meaningfully supporting long-term academic progress, reduction of underachievement, or the realization of latent potential.

Recent reviews of AI-driven personalized learning and adaptive platforms repeatedly note that long-term educational impact remains underexplored relative to immediate performance gains (Fortuna et al., 2025; Tan et al., 2025; Hariyanto et al., 2025). This challenge is especially salient for the present study because the concept of unlocking students' potential requires a developmental perspective. A meaningful personalized intervention framework should therefore be evaluated not only by local prediction quality, but also by whether it contributes to longer-term academic improvement.

#### 2.2.4 Interpretability, equity, and implementation constraints

A fourth challenge involves interpretability, equity, and real-world implementation. Reviews of AI in education consistently highlight concerns about data privacy, ethical governance, infrastructure limitations, teacher readiness, scalability, and unequal access across educational contexts (Wang et al., 2024a; Tan et al., 2025; Fortuna et al., 2025). In addition, many high-performing models remain difficult to interpret pedagogically. A system may produce accurate predictions without making clear which learner conditions or intervention principles underlie its decisions.

This is particularly problematic in psychology-oriented educational research, where it is not sufficient to demonstrate that a model works; researchers must also explain why it works and how its recommendations relate to theoretically meaningful learner processes. Without such interpretability, it becomes difficult to connect computational findings with educational practice or psychological theory. Furthermore, if AI systems are deployed without careful attention to equity and contextual constraints, personalization may inadvertently reproduce existing inequalities rather than reduce them (Dumont and Ready, 2023; Brummelman et al., 2024).

### 2.3 Research gap and positioning of the present study

Taken together, the literature suggests that AI-driven personalized learning has advanced considerably, but still faces a key conceptual gap. Existing research has shown that adaptive learning can improve outcomes, that knowledge tracing can strengthen learner diagnosis, that academic prediction can identify risk, and that AI-generated feedback can provide timely support (Contrino et al., 2024; Shen et al., 2024; Na Nongkhai et al., 2026; Wang and Luo, 2024). However, these achievements remain insufficiently integrated at the level of mechanism-aware intervention. In particular, the current literature does not yet adequately explain how learner diagnosis, intervention-relevant mechanisms, personalized action selection, and dual-horizon academic outcomes can be linked within a single coherent framework.

The present study addresses this gap by proposing MAPLe-I, a **Mechanism-Aware Personalized Learning Intervention** framework. Unlike prior approaches that focus separately on adaptive delivery, feedback generation, learner modeling, or academic prediction, MAPLe-I integrates four core components: learner-state encoding, latent mechanism estimation, intervention policy learning, and dual-horizon outcome modeling. In doing so, the proposed framework positions personalized learning not merely as adaptive content allocation, but as a mechanism-sensitive intervention process. This positioning directly responds to the shortcomings identified in the literature and establishes the conceptual basis for the methodological design presented in the next chapter.

### 2.4 Research questions and theoretical focus

Based on the literature reviewed above, the present study conceptualizes AI-driven personalized learning intervention as a mechanism-sensitive process in which learner-state diagnosis, mechanism estimation, and intervention policy jointly shape academic improvement. Rather than testing a set of isolated hypotheses, this study focuses on a group of research questions that align more directly with the design and evaluation of the proposed MAPLe-I framework.

**RQ1**. Can AI-driven personalized learning intervention improve both short-term learning outcomes and long-term academic outcomes?

**RQ2**. To what extent can engagement-related state, self-regulation-related readiness, and affective support need serve as meaningful mechanism variables for characterizing learner conditions in personalized intervention?

**RQ3**. Does a mechanism-aware intervention policy provide better pedagogical alignment than approaches that rely primarily on observable performance history or single-task optimization?

**RQ4**. Can the integration of learner-state encoding, latent mechanism estimation, intervention policy learning, and dual-horizon outcome modeling provide a more effective account of academic improvement than existing educational AI approaches that treat these components separately?

Taken together, these research questions reflect a mechanism-based view of academic improvement. Specifically, the study assumes that the educational value of AI-driven personalized intervention lies not only in adaptive instructional adjustment, but also in its ability to infer psychologically meaningful learner conditions and to align pedagogical responses with learners' engagement, self-regulation-related readiness, and affective support needs over time.

For conceptual clarity, two terms are used in a deliberately constrained sense throughout this paper. First, *mechanism-aware* does not mean that MAPLe-I directly measures or discovers validated psychological mechanisms; rather, it means that intervention decisions are conditioned on theory-informed latent proxies that are intended to approximate intervention-relevant learner conditions. Second, *unlocking students' potential* is used as an operational shorthand for identifying learners whose model-estimated longer-horizon outcomes exceed their observed baseline performance under the benchmark protocol; it should not be read as an independently validated developmental diagnosis.

## 3 Method

This chapter presents the proposed **MAPLe-I** (**M**echanism-**A**ware **P**ersonalized **Le**arning **I**ntervention) framework. The model was developed to address a central limitation in existing educational AI research: most prior approaches focus on only one aspect of intelligent support, such as adaptive delivery, feedback generation, knowledge tracing, or academic outcome prediction, whereas academic improvement typically emerges from the coordinated functioning of learner-state diagnosis, psychologically meaningful mechanism estimation, intervention decision-making, and longitudinal optimization.

Accordingly, MAPLe-I was designed as a unified and intervention-oriented framework that links learner-state representation, latent mechanism estimation, personalized intervention policy learning, and dual-horizon academic outcome modeling within a single architecture. This design is especially aligned with the theoretical objective of the present study, namely, to examine how AI-driven personalized learning interventions can unlock students' potential and improve academic achievement through interpretable underlying mechanisms.

### 3.1 Research objective and problem formulation

Let learner *i*∈{1, 2, …, *N*} interact with a digital learning environment over a sequence of time steps *t*∈{1, 2, …, *T*_*i*_}. At each time step, the learner generates an interaction record containing behavioral, knowledge-related, affect-related proxy, and contextual information. The goal of MAPLe-I is to learn a personalized intervention function that maps the learner's evolving state to the most appropriate support action while simultaneously estimating the mechanism variables associated with academic improvement.

The input vector for learner *i* at step *t* is defined as


$$\begin{array}{l} {\text{x}}_{i,t}=\left[{\text{b}}_{i,t};{\text{k}}_{i,t};{\text{e}}_{i,t};{\text{c}}_{i}\right], \end{array}$$ (1)

where:

- **b**_*i, t*_ denotes behavioral features, including response correctness, response time, hint usage, retry count, and interaction statistics;
- **k**_*i, t*_ denotes knowledge-related features, including item identifiers, concept tags, and concept dependency information;
- **e**_*i, t*_ denotes affect- or engagement-related observable proxy features;
- **c**_*i*_ denotes learner-level contextual information, if available.

The personalized intervention policy is formulated as


$$\begin{array}{l} \pi_{\theta }:{\text{x}}_{i,1:t}\mapsto {\text{a}}_{i,t}, \end{array}$$ (2)

where **a**_*i, t*_ denotes the intervention action at time step *t*, and θ denotes the trainable parameters of the policy.

The intervention action is represented as


$$\begin{array}{l} {\text{a}}_{i,t}=\left[a_{i,t}^{\left(f\right)},a_{i,t}^{\left(h\right)},a_{i,t}^{\left(d\right)},a_{i,t}^{\left(r\right)},a_{i,t}^{\left(p\right)}\right], \end{array}$$ (3)

where $a_{i,t}^{\left(f\right)}$ denotes feedback selection, $a_{i,t}^{\left(h\right)}$ denotes hint allocation, $a_{i,t}^{\left(d\right)}$ denotes difficulty adjustment, $a_{i,t}^{\left(r\right)}$ denotes remedial support, and $a_{i,t}^{\left(p\right)}$ denotes pacing control.

In addition, the framework estimates a latent mechanism vector:


$$\begin{array}{l} {\text{m}}_{i,t}=\left[m_{i,t}^{\left(eng\right)},m_{i,t}^{\left(srl\right)},m_{i,t}^{\left(aff\right)}\right], \end{array}$$ (4)

where $m_{i,t}^{\left(eng\right)}$ denotes the engagement-related mechanism score, $m_{i,t}^{\left(srl\right)}$ denotes the self-regulation-related readiness score, and $m_{i,t}^{\left(aff\right)}$ denotes the affective support need score.

For conceptual precision, these three quantities are modeled as theory-informed computational proxies rather than as validated psychological measurements. In substantive terms, $m_{i,t}^{\left(eng\right)}$ is intended to approximate active task involvement, $m_{i,t}^{\left(srl\right)}$ approximates readiness for sustained self-regulated learning control, and $m_{i,t}^{\left(aff\right)}$ approximates the likely need for supportive affect-sensitive intervention. Their interpretation therefore remains provisional and should ultimately be validated against psychometric or observational evidence in future work.

Finally, MAPLe-I predicts both short-term and long-term outcomes:


$$\begin{array}{l} {ŷ}_{i,t}^{\left(short\right)}=f_{s}\left({\text{x}}_{i,1:t},{\text{a}}_{i,t}\right), \end{array}$$ (5)

$$\begin{array}{l} {ŷ}_{i}^{\left(long\right)}=f_{l}\left({\text{x}}_{i,1:T_{i}},{\text{a}}_{i,1:T_{i}}\right), \end{array}$$ (6)

where ${ŷ}_{i,t}^{\left(short\right)}$ denotes the short-term learning outcome and ${ŷ}_{i}^{\left(long\right)}$ denotes the long-term academic outcome.

The overall optimization problem is formulated as


$$\begin{array}{l} \theta^{*}=\arg\underset{\theta }{\min}L\left({ŷ}^{\left(short\right)},{ŷ}^{\left(long\right)},\text{m},\text{a}\right), \end{array}$$ (7)

where L(·) jointly accounts for short-term prediction, long-term prediction, mechanism estimation, and intervention policy learning.

### 3.2 Notation Summary

For clarity, the principal notations used in this chapter are summarized in Table 1.

**Table 1:** Principal notations used in the MAPLe-I framework.

Symbol	Description
*N*	Total number of learners.
*T*_*i*_	Total number of valid interaction steps for learner *i*.
**x**_*i, t*_	Input feature vector of learner *i* at time step *t*.
**b**_*i, t*_	Behavioral feature vector.
**k**_*i, t*_	Knowledge-related feature vector.
**e**_*i, t*_	Affect- or engagement-related observable proxy vector.
**c**_*i*_	Learner-level contextual feature vector.
G=(V,E)	Knowledge graph consisting of concepts and their relations.
**z**_*i, t*_	Latent learner-state representation.
**m**_*i, t*_	Estimated mechanism vector.
${\tilde{\text{z}}}_{i,t}$	Mechanism-enhanced learner-state representation.
**a**_*i, t*_	Personalized intervention action.
${ŷ}_{i,t}^{\left(short\right)}$	Predicted short-term learning outcome.
${ŷ}_{i}^{\left(long\right)}$	Predicted long-term academic outcome.
Δ_*i*_	Potential-improvement index.
π_θ_	Intervention policy network parameterized by θ.
$L_{short}$	Short-term outcome loss.
$L_{long}$	Long-term outcome loss.
$L_{mech}$	Mechanism estimation or regularization loss.
$L_{policy}$	Intervention policy learning loss.
L	Overall training objective.

### 3.3 Overview of MAPLe-I

**Figure 1:**
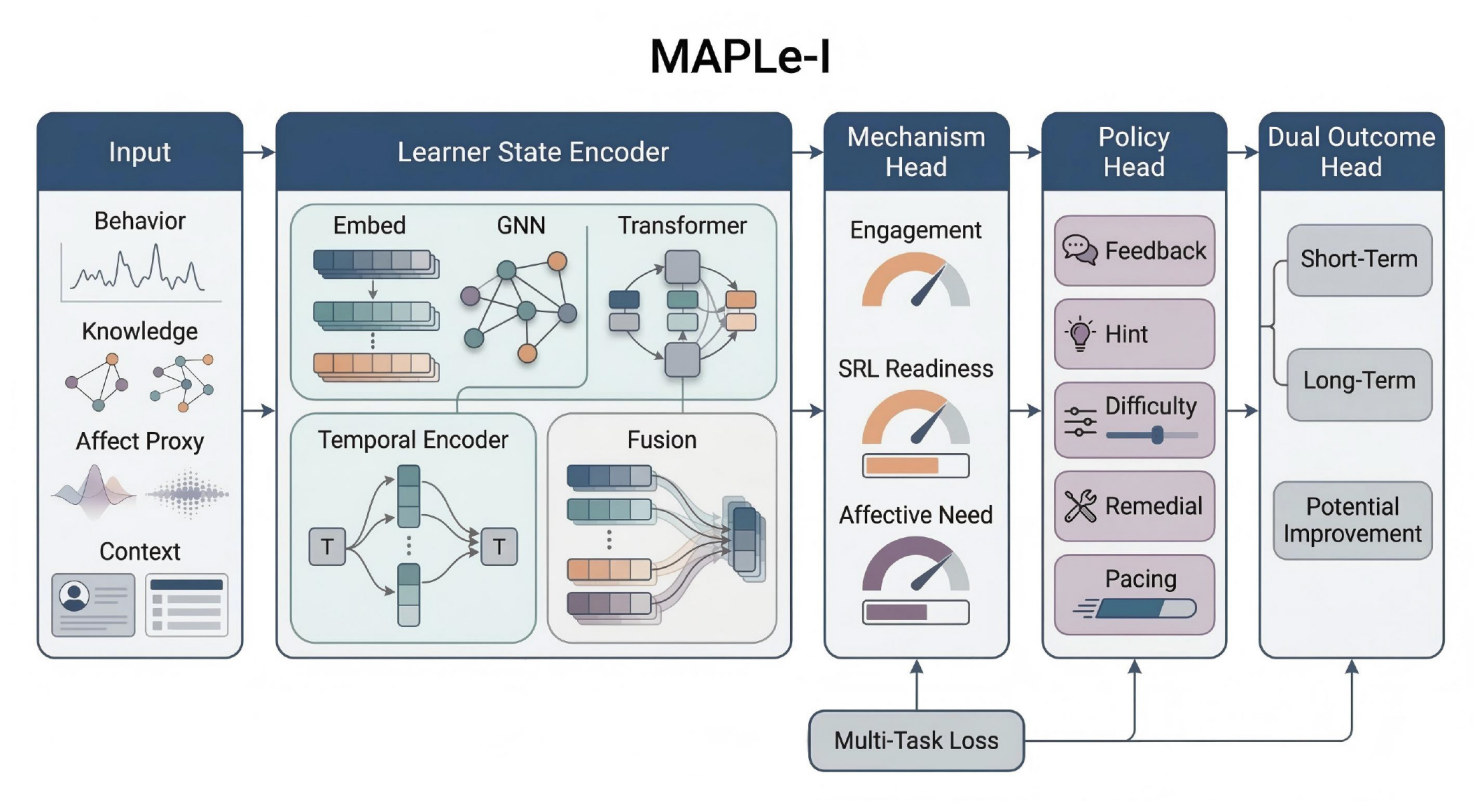
Overview of the proposed MAPLe-I framework. MAPLe-I integrates multi-source learner inputs, learner-state encoding, mechanism estimation, personalized intervention policy learning, and dual-horizon academic outcome modeling. Specifically, behavioral, knowledge, affect-related proxy, and contextual features are first encoded into a latent learner-state representation, which is then used to estimate engagement, self-regulation-related readiness, and affective support need. Based on these mechanism-aware representations, the policy head generates personalized support actions, and the dual outcome head jointly predicts short-term and long-term academic outcomes, together with potential improvement.

As illustrated in Figure 1, MAPLe-I is organized as a unified mechanism-aware personalized learning framework that integrates learner-state encoding, latent mechanism estimation, intervention policy learning, and dual-horizon academic outcome modeling.

MAPLe-I consists of four functionally coordinated modules:

1. a **Learner State Encoder**,
2. a **Mechanism Estimation Head**,
3. an **Intervention Policy Head**,
4. and a **Dual Outcome Head**.

The general computation pipeline is


$$\begin{array}{l} {\text{z}}_{i,t}=f_{\text{enc}}\left({\text{x}}_{i,1:t}\right), \end{array}$$ (8)

$$\begin{array}{l} {\text{m}}_{i,t}=f_{\text{mech}}\left({\text{z}}_{i,t}\right), \end{array}$$ (9)

$$\begin{array}{l} {\text{a}}_{i,t}=\pi_{\theta }\left({\text{z}}_{i,t},{\text{m}}_{i,t}\right), \end{array}$$ (10)

$$\begin{array}{l} {ŷ}_{i,t}^{\left(short\right)}=f_{s}\left({\text{z}}_{i,t},{\text{m}}_{i,t},{\text{a}}_{i,t}\right), \end{array}$$ (11)

$$\begin{array}{l} {ŷ}_{i}^{\left(long\right)}=f_{l}\left({\text{z}}_{i,1:T_{i}},{\text{m}}_{i,1:T_{i}},{\text{a}}_{i,1:T_{i}}\right). \end{array}$$ (12)

This architecture operationalizes the following working theoretical chain:


$$\begin{array}{l} \text{Learner State}\to \text{Mechanism Estimation}\to \end{array}$$

$$\begin{array}{l} \text{Personalized Intervention}\to \text{Academic Improvement}. \end{array}$$ (13)

### 3.4 Variable definitions

Table 2 presents the operational definitions of the key variables in MAPLe-I.

**Table 2:** Operational definitions of the key variables in MAPLe-I.

Variable	Type	Operational definition
Behavioral features	Input variable	Interaction-level features including correctness, response time, hint usage, retry count, and related behavioral indicators.
Knowledge features	Input variable	Item and concept features, including concept tags and dependency structure.
Affect/engagement proxies	Input variable	Observable affective or engagement-related indicators, such as boredom, confusion, frustration, or behavior-derived proxy variables.
Contextual features	Input variable	Learner-level background information, such as demographic or course-related attributes.
Learner state	Latent variable	A fused representation of current performance, concept mastery, and interaction trajectory.
Engagement-related mechanism	Latent proxy variable	A computationally estimated proxy representing the learner's active cognitive-behavioral involvement; it is not a validated psychometric score.
Self-regulation-related readiness	Latent proxy variable	A computationally estimated proxy indicating readiness for sustained and adaptive learning control rather than a direct psychological assessment.
Affective support need	Latent proxy variable	A computationally estimated proxy indicating the extent to which the learner may require motivational or emotional support.
Feedback decision	Action variable	The model's selection of feedback type.
Hint allocation	Action variable	The model's decision regarding hint support.
Difficulty adjustment	Action variable	The model's decision to increase, maintain, or reduce task difficulty.
Remedial support	Action variable	The model's decision to recommend review or supplementary practice.
Pacing control	Action variable	The model's decision regarding progression speed.
Short-term learning outcome	Outcome variable	Immediate learning performance, such as next-response correctness or local mastery gain.
Long-term academic outcome	Outcome variable	Broader academic status under the benchmark setting, such as stage performance or course achievement; it is not itself evidence of causal developmental change.
Potential-improvement index	Derived descriptor	A model-based descriptor of predicted gain relative to learner baseline performance; it is not an independently validated measure of unrealized potential.

### 3.5 Learner state encoder

The learner state encoder is responsible for transforming heterogeneous learner data into a compact latent representation. Because educational interaction data are both sequential and concept-structured, a graph-temporal encoding strategy is adopted.

#### 3.5.1 Behavioral feature representation

At each time step, learner behavior is represented as


$$\begin{array}{l} {\text{b}}_{i,t}=\left[r_{i,t},\tau_{i,t},h_{i,t},q_{i,t},s_{i,t}\right], \end{array}$$ (14)

where *r*_*i, t*_ denotes response correctness, τ_*i, t*_ denotes response time, *h*_*i, t*_ denotes hint usage, *q*_*i, t*_ denotes retry count, and *s*_*i, t*_ denotes additional interaction statistics.

The raw behavioral vector is projected into a dense embedding:


$$\begin{array}{l} {\text{u}}_{i,t}^{\left(b\right)}={\text{W}}_{b}{\text{b}}_{i,t}+{\text{b}}_{b}, \end{array}$$ (15)

where **W**_*b*_ and **b**_*b*_ are trainable parameters.

#### 3.5.2 Knowledge structure representation

Let G=(V,E) denote the knowledge graph, where V is the concept set and E denotes the relations among concepts. The graph encoder updates concept representations through message passing:


$$\begin{array}{l} {\text{h}}_{v}^{\left(g,l+1\right)}=\sigma \left(\underset{u\in N\left(v\right)}{\sum }\alpha_{uv}^{\left(l\right)}{\text{W}}_{g}^{\left(l\right)}{\text{h}}_{u}^{\left(g,l\right)}\right), \end{array}$$ (16)

where N(v) denotes the neighbor set of concept *v*, $\alpha_{uv}^{\left(l\right)}$ denotes the aggregation weight, and ${\text{W}}_{g}^{\left(l\right)}$ denotes the trainable transformation matrix.

The graph-based concept representation is


$$\begin{array}{l} {\text{u}}_{i,t}^{\left(g\right)}=\text{GNN}\left(G,{\text{k}}_{i,t}\right). \end{array}$$ (17)

#### 3.5.3 Affect-related proxy representation

To incorporate affect- or engagement-related observable proxy information into the learner-state representation, the proxy vector is projected into a dense embedding space:


$$\begin{array}{l} {\text{u}}_{i,t}^{\left(e\right)}={\text{W}}_{e}{\text{e}}_{i,t}+{\text{b}}_{e}, \end{array}$$ (18)

where **W**_*e*_ and **b**_*e*_ are trainable parameters.

#### 3.5.4 Temporal sequence encoding

The fused interaction token at each step is defined as


$$\begin{array}{l} {\text{u}}_{i,t}=\left[{\text{u}}_{i,t}^{\left(b\right)};{\text{u}}_{i,t}^{\left(g\right)};{\text{u}}_{i,t}^{\left(e\right)};{\text{c}}_{i}\right], \end{array}$$ (19)

The temporal input sequence is then represented as


$$\begin{array}{l} {\text{U}}_{i,1:t}=\left[{\text{u}}_{i,1},{\text{u}}_{i,2},\ldots ,{\text{u}}_{i,t}\right]. \end{array}$$ (20)

A Transformer-style encoder is used to capture temporal dependency:


$$\begin{array}{l} \text{Attention}\left(\text{Q},\text{K},\text{V}\right)=\text{softmax}\left(\frac{\text{Q}{\text{K}}^{⊤}}{\sqrt{d_{k}}}\right)\text{V}, \end{array}$$ (21)

where **Q**, **K**, and **V** denote the query, key, and value matrices, respectively.

The temporal learner representation is


$$\begin{array}{l} {\text{u}}_{i,t}^{\left(s\right)}=\text{SeqEnc}\left({\text{U}}_{i,1:t}\right). \end{array}$$ (22)

#### 3.5.5 Cross-modal fusion

The final latent learner-state representation is obtained by fusing graph-based and temporal representations:


$$\begin{array}{l} {\text{z}}_{i,t}=\phi \left({\text{W}}_{z}\left[{\text{u}}_{i,t}^{\left(g\right)};{\text{u}}_{i,t}^{\left(s\right)};{\text{c}}_{i}\right]+{\text{b}}_{z}\right), \end{array}$$ (23)

where ϕ(·) is a nonlinear activation function and **W**_*z*_, **b**_*z*_ are trainable parameters.

### 3.6 Mechanism estimation head

A defining feature of MAPLe-I is the explicit estimation of latent intervention-relevant proxy variables. Rather than assuming that academic improvement is fully explained by observable performance alone, the model estimates theory-informed latent states associated with engagement, self-regulation-related readiness, and affective support need. In this paper, these quantities are treated as computational approximations that may help organize intervention decisions, not as direct measurements of validated psychological mechanisms.

#### 3.6.1 Mechanism inference

The mechanism vector is computed as


$$\begin{array}{l} {\text{m}}_{i,t}=\sigma_{m}\left({\text{W}}_{m}{\text{z}}_{i,t}+{\text{b}}_{m}\right), \end{array}$$ (24)

where **W**_*m*_ and **b**_*m*_ are trainable parameters and σ_*m*_(·) denotes the activation function.

The three mechanism scores are


$$\begin{array}{l} m_{i,t}^{\left(eng\right)}=\sigma \left({\text{w}}_{eng}^{⊤}{\text{z}}_{i,t}+b_{eng}\right), \end{array}$$ (25)

$$\begin{array}{l} m_{i,t}^{\left(srl\right)}=\sigma \left({\text{w}}_{srl}^{⊤}{\text{z}}_{i,t}+b_{srl}\right), \end{array}$$ (26)

$$\begin{array}{l} m_{i,t}^{\left(aff\right)}=\sigma \left({\text{w}}_{aff}^{⊤}{\text{z}}_{i,t}+b_{aff}\right). \end{array}$$ (27)

These values are interpreted as latent mechanism proxies rather than direct psychometric measurements. Accordingly, any explanatory claims made later in the paper should be understood as model-based interpretations of intervention-relevant learner conditions, not as definitive evidence of mechanism discovery.

#### 3.6.2 Mechanism-aware state enhancement

To ensure that subsequent intervention decisions are sensitive to these latent states, the model constructs a mechanism-enhanced representation:


$$\begin{array}{l} {\tilde{\text{z}}}_{i,t}=\left[{\text{z}}_{i,t};{\text{m}}_{i,t}\right]. \end{array}$$ (28)

### 3.7 Intervention policy head

The intervention policy head transforms learner-state diagnosis into actionable educational support.

#### 3.7.1 Action prediction

The intervention policy is defined over the mechanism-enhanced learner-state representation:


$$\begin{array}{l} {\text{a}}_{i,t}=\left[\pi^{\left(f\right)}\left({\tilde{\text{z}}}_{i,t}\right),\pi^{\left(h\right)}\left({\tilde{\text{z}}}_{i,t}\right),\pi^{\left(d\right)}\left({\tilde{\text{z}}}_{i,t}\right),\pi^{\left(r\right)}\left({\tilde{\text{z}}}_{i,t}\right),\pi^{\left(p\right)}\left({\tilde{\text{z}}}_{i,t}\right)\right], \end{array}$$ (29)

where each π^(·)^(·) denotes an action-specific prediction head.

For a generic action head, the action probability distribution is computed as


$$\begin{array}{l} P\left(a|{\tilde{\text{z}}}_{i,t}\right)=\text{softmax}\left({\text{W}}_{a}{\tilde{\text{z}}}_{i,t}+{\text{b}}_{a}\right), \end{array}$$ (30)

where **W**_*a*_ and **b**_*a*_ are trainable parameters.

The selected intervention is


$$\begin{array}{l} a_{i,t}^{*}=\arg\underset{a\in A}{\max}P\left(a|{\tilde{\text{z}}}_{i,t}\right), \end{array}$$ (31)

where A denotes the admissible action set.

#### 3.7.2 Rule-based construction of intervention labels

Because the public datasets used in this study do not provide native teacher- or system-recorded intervention labels for feedback type, hint intensity, difficulty adjustment, remedial recommendation, or pacing control, the supervision targets for the intervention policy were constructed offline using a pedagogically grounded rule base. Accordingly, the intervention labels used in this study should be interpreted as *rule-derived pedagogical pseudo-labels* rather than directly observed classroom interventions. This design supports a transparent first-stage benchmark, but it also places an important boundary on how the intervention-policy results should be interpreted.

The rule schema was defined *a priori* by the author on the basis of educational psychology principles concerning scaffolding, adaptive challenge, process-oriented feedback, affect-sensitive support, and self-regulated learning support. The purpose of this rule base was to translate learner-state diagnosis and mechanism-proxy estimates into interpretable pedagogical action targets that are consistent with the theoretical positioning of MAPLe-I as a mechanism-aware intervention framework. Importantly, this procedure does not validate authentic intervention quality in real educational settings; instead, it evaluates whether the policy head can reproduce a theoretically specified rule system on held-out data.

Let ${\bar{r}}_{i,t}$ denote recent correctness, κ_*i, t*_ denote concept-mastery level, ${\bar{h}}_{i,t}$ denote recent hint dependence, ${\bar{q}}_{i,t}$ denote retry intensity, $\tau_{i,t}^{\left(z\right)}$ denote the standardized response time, and $m_{i,t}^{\left(eng\right)}$, $m_{i,t}^{\left(srl\right)}$, and $m_{i,t}^{\left(aff\right)}$ denote the three mechanism scores. The five intervention dimensions were defined as follows:

- **Feedback selection** ($a_{i,t}^{\left(f\right)}$): supportive feedback was assigned when affective support need was high; strategy-oriented feedback was assigned when self-regulation-related readiness was low; explanatory feedback was assigned when correctness or mastery was low but affective support need was not dominant; affirming feedback was assigned when performance was adequate and the learner mainly required reinforcement or confidence support.
- **Hint allocation** ($a_{i,t}^{\left(h\right)}$): no hint was assigned when mastery and self-regulation-related readiness were both high; light hints were assigned when learners showed moderate uncertainty; stepwise hints were assigned when repeated errors, high hint dependence, or low mastery indicated a need for stronger scaffolding.
- **Difficulty adjustment** ($a_{i,t}^{\left(d\right)}$): difficulty was increased when mastery, engagement, and self-regulation-related readiness were high and affective support need was low; difficulty was decreased when learners showed persistent failure, low engagement, or elevated affective support need; otherwise difficulty was maintained.
- **Remedial support** ($a_{i,t}^{\left(r\right)}$): review or targeted practice was assigned when concept mastery remained low across recent interactions or when repeated errors suggested unresolved misconceptions; otherwise no remedial action was assigned.
- **Pacing control** ($a_{i,t}^{\left(p\right)}$): pacing was slowed when learners exhibited long response times, repeated retries, high affective support need, or substantial scaffold dependence; pacing was accelerated when mastery and engagement were both high and support need was low; otherwise pacing was maintained.

Formally, the rule-derived supervisory label is defined as


$$\begin{array}{l} a_{i,t}^{obs}=R\left(z_{i,t},m_{i,t},{\bar{r}}_{i,t},\kappa_{i,t},{\bar{h}}_{i,t},{\bar{q}}_{i,t},\tau_{i,t}^{\left(z\right)}\right), \end{array}$$ (32)

where R(·) denotes the pedagogical rule engine. The same rule schema was applied to held-out learners to construct rule-consistent target labels for evaluation:


$$\begin{array}{l} a_{i,t}^{target}=R\left(z_{i,t},m_{i,t},{\bar{r}}_{i,t},\kappa_{i,t},{\bar{h}}_{i,t},{\bar{q}}_{i,t},\tau_{i,t}^{\left(z\right)}\right). \end{array}$$ (33)

Importantly, the thresholds used in R(·) were estimated on the training split only and then fixed for validation and test evaluation. Therefore, $a_{i,t}^{obs}$ served as the supervisory signal for intervention-policy learning, whereas $a_{i,t}^{target}$ served as the held-out rule-consistent reference for Intervention Alignment Rate (IAR) evaluation. Under this setup, IAR should be interpreted as agreement with the predefined pedagogical rule system rather than as direct validation against observed teacher decision-making.

### 3.8 Dual outcome head

MAPLe-I predicts both immediate and cumulative academic effects.

#### 3.8.1 Short-term outcome prediction

The short-term outcome is computed as


$$\begin{array}{l} {ŷ}_{i,t}^{\left(short\right)}=\sigma_{s}\left({\text{W}}_{s}\left[{\tilde{\text{z}}}_{i,t};{\text{a}}_{i,t}\right]+{\text{b}}_{s}\right), \end{array}$$ (34)

where σ_*s*_(·) is an output activation function.

If the target is binary correctness,


$$\begin{array}{l} P\left(y_{i,t}^{\left(short\right)}=1|{\tilde{\text{z}}}_{i,t},{\text{a}}_{i,t}\right)={ŷ}_{i,t}^{\left(short\right)}. \end{array}$$ (35)

#### 3.8.2 Long-term academic outcome prediction

The trajectory-level representation is aggregated as


$$\begin{array}{l} {\bar{\text{z}}}_{i}=\text{Pool}\left({\tilde{\text{z}}}_{i,1},{\tilde{\text{z}}}_{i,2},\ldots ,{\tilde{\text{z}}}_{i,T_{i}}\right), \end{array}$$ (36)

where Pool(·) denotes a pooling operator such as mean pooling or attention pooling.

The long-term academic outcome is then predicted as


$$\begin{array}{l} {ŷ}_{i}^{\left(long\right)}=\psi \left({\text{W}}_{l}{\bar{\text{z}}}_{i}+{\text{b}}_{l}\right), \end{array}$$ (37)

where ψ(·) is the output function for the long-term task.

#### 3.8.3 Potential-improvement index

To characterize model-estimated room for improvement under the benchmark protocol, an auxiliary *Potential-Improvement Index* is defined as


$$\begin{array}{l} \Delta_{i}={ŷ}_{i}^{\left(long\right)}-y_{i}^{\left(base\right)}, \end{array}$$ (38)

where $y_{i}^{\left(base\right)}$ denotes the learner's baseline or expected outcome level. This quantity is a model-based descriptor derived from predicted long-term outcomes relative to baseline; it is not an independently validated indicator of unrealized potential.

#### 3.8.4 Operationalization of underachievement and subgroup construction

To make the notion of “unlocking students' potential” operationally explicit without overstating its empirical status, this study treated underachievement as a benchmark mismatch between currently realized performance and model-estimated longer-horizon improvement under the proposed protocol. Let $y_{i}^{\left(base\right)}$ denote learner *i*'s baseline achievement and ${ŷ}_{i}^{\left(long\right)}$ denote the predicted long-term academic outcome. Following Equation 36, the potential-improvement index is defined as


$$\begin{array}{l} \Delta_{i}={ŷ}_{i}^{\left(long\right)}-y_{i}^{\left(base\right)}. \end{array}$$ (39)

For the sequential datasets (ASSISTments and EdNet-KT1), $y_{i}^{\left(base\right)}$ was defined as the mean correctness over the first 20% of valid interactions. For OULAD, $y_{i}^{\left(base\right)}$ was defined using early-course performance indicators available before the midpoint of the course, including early assessment results and normalized early engagement features. To avoid information leakage, all subgroup thresholds were estimated on the training set only and then applied unchanged to the validation and test sets.

Based on the joint distribution of baseline achievement and potential-improvement scores, learners were divided into three theoretically relevant subgroups:


$$\begin{array}{l} g_{i}=\\ \{\begin{array}{ll} at-risk, & \text{if }y_{i}^{\left(base\right)}\leq Q_{30}(y^{\left(base\right)})\text{ and }{\hat{y}}_{i}^{\left(long\right)}\leq Q_{30}({\hat{y}}^{\left(long\right)}),\\ underachieving, & \text{if }y_{i}^{\left(base\right)}\leq Q_{30}(y^{\left(base\right)})\text{ and }\Delta_{i}\geq Q_{70}(\Delta ),\\ low-potential-gap, & \text{if }|\Delta_{i}|\leq Q_{30}(|\Delta |),\\ general-progress, & \text{otherwise}. \end{array} \end{array}$$ (40)

The *underachieving* subgroup captures learners whose currently realized performance is relatively low while their estimated developmental room is comparatively high. The *at-risk* subgroup captures learners who are low both in current achievement and in predicted long-term outcomes. The *low-potential-gap* subgroup captures learners whose current achievement is already close to their estimated long-term trajectory. This grouping strategy enables subgroup-level validation of whether mechanism-aware intervention is especially beneficial for learners whose current performance does not fully reflect their developmental capacity.

### 3.9 Training objective

MAPLe-I is trained in a multi-task learning framework.

#### 3.9.1 Short-term outcome loss

For binary short-term targets, the short-term loss is


$$\begin{array}{l} {ℒ}_{short}=-\frac{1}{\sum_{i}T_{i}}\sum_{i=1}^{N}\sum_{t=1}^{T_{i}}[y_{i,t}^{\left(short\right)}\log{\hat{y}}_{i,t}^{\left(short\right)}\\ \;+(1-y_{i,t}^{\left(short\right)})\log(1-{\hat{y}}_{i,t}^{\left(short\right)})]. \end{array}$$ (41)

#### 3.9.2 Long-term academic outcome loss

For continuous long-term outcomes, the loss is


$$\begin{array}{l} L_{long}=\frac{1}{N}\overset{N}{\underset{i=1}{\sum }}{\left(y_{i}^{\left(long\right)}-{ŷ}_{i}^{\left(long\right)}\right)}^{2}. \end{array}$$ (42)

For categorical outcomes, cross-entropy can be used:


$$\begin{array}{l} L_{long}^{\left(ce\right)}=-\frac{1}{N}\overset{N}{\underset{i=1}{\sum }}\overset{C}{\underset{c=1}{\sum }}I\left(y_{i}=c\right)\log{\hat{p}}_{i,c}. \end{array}$$ (43)

#### 3.9.3 Mechanism loss

Mechanism learning is regularized as


$$\begin{array}{l} L_{mech}=\frac{1}{\sum_{i}T_{i}}\overset{N}{\underset{i=1}{\sum }}\overset{T_{i}}{\underset{t=1}{\sum }}||{\text{m}}_{i,t}-{\hat{\text{m}}}_{i,t}^{\left(proxy\right)}{||}_{2}^{2}, \end{array}$$ (44)

where ${\hat{\text{m}}}_{i,t}^{\left(proxy\right)}$ denotes proxy-derived target values when available.

When direct proxy targets are unavailable, temporal smoothness is imposed:


$$\begin{array}{l} L_{smooth}=\frac{1}{\sum_{i}\left(T_{i}-1\right)}\overset{N}{\underset{i=1}{\sum }}\overset{T_{i}}{\underset{t=2}{\sum }}||{\text{m}}_{i,t}-{\text{m}}_{i,t-1}{||}_{2}^{2}. \end{array}$$ (45)

#### 3.9.4 Intervention policy loss

The policy loss is defined as


$$\begin{array}{l} L_{policy}=-\frac{1}{\sum_{i}T_{i}}\overset{N}{\underset{i=1}{\sum }}\overset{T_{i}}{\underset{t=1}{\sum }}\sum_{a\in A}I\left(a=a_{i,t}^{obs}\right)\log P\left(a|{\tilde{\text{z}}}_{i,t}\right), \end{array}$$ (46)

where $a_{i,t}^{obs}$ denotes the observed or target intervention label.

In this study, $a_{i,t}^{obs}$ was not directly observed from the raw datasets but was constructed offline using the pedagogical rule base described in Section 3.7.2.

#### 3.9.5 Overall objective

The overall objective is


$$\begin{array}{l} L=\lambda_{1}L_{short}+\lambda_{2}L_{long}+\lambda_{3}L_{mech}+\lambda_{4}L_{policy}+\lambda_{5}||\Theta {||}_{2}^{2}, \end{array}$$ (47)

where Θ denotes the full set of trainable parameters.

The optimal parameters are obtained by


$$\begin{array}{l} \Theta^{*}=\arg\underset{\Theta }{\min}L. \end{array}$$ (48)

### 3.10 Datasets

A multi-dataset design was adopted because no single publicly available dataset simultaneously captures fine-grained interaction processes, affective proxies, large-scale sequential behavior, and longer-horizon academic outcomes in a fully integrated manner. The three datasets were therefore selected for complementary methodological roles rather than as interchangeable benchmarks. Together, they allow the proposed framework to be examined across interaction-level learner-state modeling, external sequential generalization, and longer-horizon academic outcome prediction.

#### 3.10.1 ASSISTments 2012–2013 with affect

ASSISTments 2012–2013 with Affect was used as the primary dataset (Heffernan and Heffernan, 2014). For learner *i*, the sequence is represented as


$$\begin{array}{l} S_{i}^{A}=\left\{\left({\text{b}}_{i,1},{\text{k}}_{i,1},{\text{e}}_{i,1}\right),\ldots ,\left({\text{b}}_{i,T_{i}},{\text{k}}_{i,T_{i}},{\text{e}}_{i,T_{i}}\right)\right\}. \end{array}$$ (49)

This dataset was chosen as the primary benchmark because it provides the richest alignment with the full MAPLe-I pipeline: interaction-level sequences, knowledge-linked practice records, and affect-related observations that can be used as proxy inputs. It is therefore the only dataset in the present study that supports joint analysis of learner-state encoding, latent proxy estimation, rule-derived intervention alignment, and short-term outcome prediction within a single benchmark setting.

#### 3.10.2 EdNet-KT1

EdNet-KT1 was used as an external validation dataset for large-scale learner-state modeling and generalization analysis (Choi et al., 2020b):


$$\begin{array}{l} S_{i}^{E}=\left\{\left({\text{b}}_{i,1},{\text{k}}_{i,1}\right),\ldots ,\left({\text{b}}_{i,T_{i}},{\text{k}}_{i,T_{i}}\right)\right\}. \end{array}$$ (50)

EdNet-KT1 was selected because of its very large scale and dense sequential interaction structure. Although it does not provide the same affect-related richness as ASSISTments, it is well suited to testing whether the learner-state encoder and the broader modeling strategy retain strong performance in a substantially larger and behaviorally diverse environment. In other words, EdNet-KT1 primarily serves as an external generalization benchmark rather than as a full intervention-quality benchmark.

#### 3.10.3 OULAD

OULAD was used for long-term academic outcome evaluation (Kuzilek et al., 2017):


$$\begin{array}{l} {\text{x}}_{i}^{O}=\left[{\text{c}}_{i};{\text{v}}_{i};{\text{g}}_{i}\right], \end{array}$$ (51)

where **v**_*i*_ denotes virtual learning environment interaction features and **g**_*i*_ denotes assessment-related outcome features.

OULAD was selected because it offers course-level academic outcomes and rich virtual-learning-environment traces that are more appropriate for longer-horizon academic modeling than the item-level datasets alone. It therefore complements ASSISTments and EdNet-KT1 by providing a benchmark for the long-term outcome head and for the model-based Potential-Improvement Index. Taken together, the three datasets support complementary but not identical forms of evaluation, which is why the manuscript reports dataset-specific roles rather than claiming that every module is tested under exactly the same observational conditions.

### 3.11 Data preprocessing

To ensure consistency across datasets, the following unified preprocessing pipeline was adopted.

#### 3.11.1 Sequence construction

For each learner, interactions were ordered chronologically:


$$\begin{array}{l} S_{i}=\left\{{\text{x}}_{i,1},{\text{x}}_{i,2},\ldots ,{\text{x}}_{i,T_{i}}\right\}. \end{array}$$ (52)

#### 3.11.2 Feature normalization and encoding

Continuous variables were standardized using z-score normalization:


$$\begin{array}{l} x^{\prime }=\frac{x-\mu }{\sigma }, \end{array}$$ (53)

where μ and σ denote the mean and standard deviation estimated from the training set.

Categorical features were mapped to embeddings:


$$\begin{array}{l} {\text{e}}_{cat}=\text{E}\left[id\right]. \end{array}$$ (54)

#### 3.11.3 Sparse sequence filtering

Learners with insufficient interaction history were excluded:


$$\begin{array}{l} D^{*}=\left\{i|T_{i}\geq T_{\min}\right\}. \end{array}$$ (55)

#### 3.11.4 Train–validation–test split

The data were partitioned at the learner level:


$$\begin{array}{l} D=D_{train}\cup D_{val}\cup D_{test}, \end{array}$$ (56)

with


$$\begin{array}{l} D_{train}\cap D_{val}=\emptyset ,\;D_{train}\cap D_{test}=\emptyset ,\;D_{val}\cap D_{test}=\emptyset . \end{array}$$ (57)

### 3.12 Algorithmic procedure

Algorithm 1 summarizes the end-to-end procedure of MAPLe-I.

Algorithm 1 **MAPLe-I: mechanism-aware personalized learning intervention.**

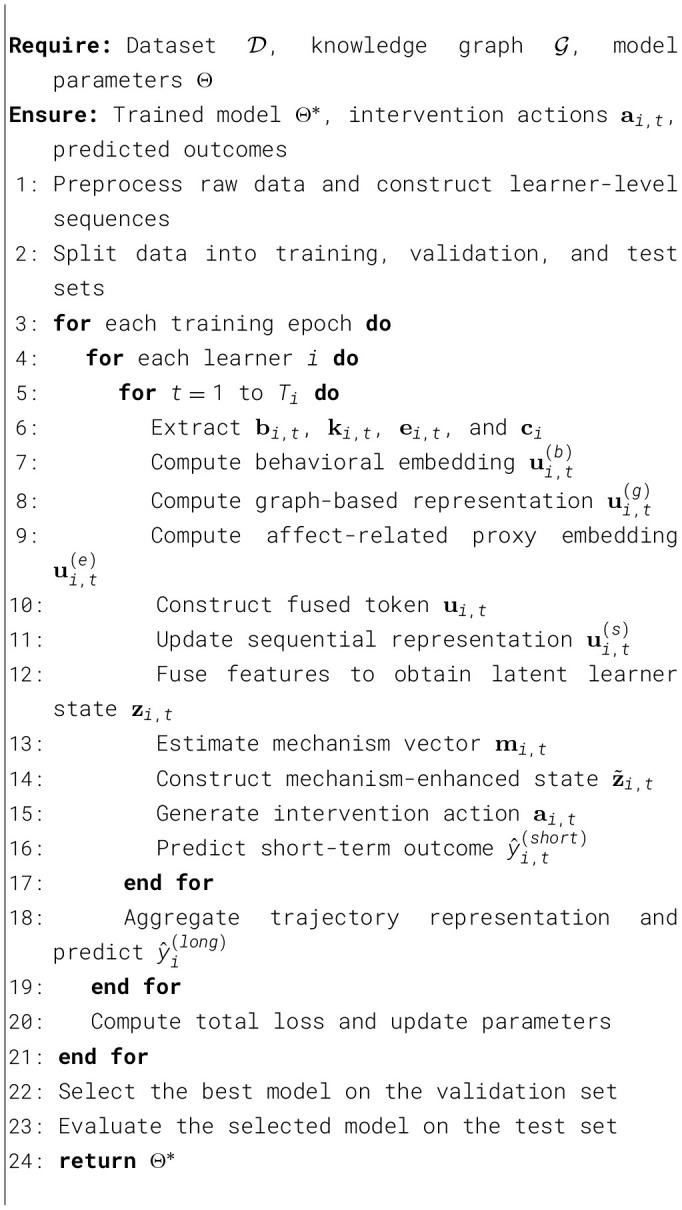

### 3.13 Chapter summary

In summary, MAPLe-I was designed as a mechanism-aware and intervention-oriented framework that integrates learner-state diagnosis, latent mechanism estimation, personalized decision-making, and dual-horizon academic outcome modeling. This chapter established the conceptual and computational foundation for the empirical evaluation reported in the next chapter.

## 4 Experiments

This chapter evaluates the proposed MAPLe-I framework from multiple complementary perspectives, including baseline comparison, metric design, experimental settings, main empirical results, and additional analyses concerning ablation, parameter sensitivity, explanation, and limitations. In line with the objective of this study, the experiments were designed not only to test whether MAPLe-I improves predictive performance, but also to examine whether a mechanism-aware personalized intervention framework can outperform conventional digital instruction, traditional adaptive learning, feedback-centered AI approaches, and learner modeling or academic prediction baselines.

In all result tables, underlined values denote the second-best results under each metric, whereas ^**^ indicates that MAPLe-I significantly outperformed the strongest corresponding baseline at *p* < 0.01.

### 4.1 Baselines

To provide a rigorous and theory-aligned evaluation, MAPLe-I was compared with four categories of baselines: a non-personalized digital intervention baseline, a traditional adaptive learning baseline, AI-based feedback baselines, and learner modeling or academic prediction baselines. This design was intended to test whether the proposed framework can deliver performance gains beyond uniform digital instruction, platform-level adaptive learning, feedback-only paradigms, and prediction-oriented methods.

#### 4.1.1 Non-personalized digital intervention

In this condition, all learners receive identical learning materials, task progression, and feedback regardless of prior performance or evolving learner state. This baseline represents a one-size-fits-all digital learning setting and serves as the most basic comparison condition for evaluating whether personalization itself contributes to benchmark performance improvements.

#### 4.1.2 Traditional adaptive learning baseline

The traditional adaptive learning baseline approximates the logic of a platform-based adaptive learning system, following the general adaptive progression principle represented by CogBooks-style learning environments (Contrino et al., 2024). In the present implementation, this baseline receives the same chronological learner records as MAPLe-I but uses rule-based progression and difficulty updating driven primarily by recent correctness and concept progression. It does not explicitly estimate intervention-relevant proxies, does not maintain a separate multi-head pedagogical policy, and does not jointly optimize short-term and long-term outcomes.

#### 4.1.3 AI-based feedback baselines

Three feedback baselines were included to evaluate whether MAPLe-I outperforms feedback-centered approaches:

- **Adaptive Feedback**, which generates feedback based on rule-based adaptive logic;
- **GenAI Feedback**, which generates feedback using a generative AI model;
- **Hybrid Feedback**, which combines adaptive logic with generative AI feedback.

These baselines follow the comparative logic of recent work on adaptive and generative-AI-based educational feedback (Na Nongkhai et al., 2026). For fairness, all three feedback baselines use the same input partitions and the same learner-level chronological records, but they differ in how pedagogical responses are produced: Adaptive Feedback uses handcrafted rule mapping from recent performance to feedback category; GenAI Feedback uses a generative feedback module conditioned on compact learner summaries without an explicit mechanism layer; and Hybrid Feedback first applies adaptive state rules and then uses a generative component to verbalize the selected feedback intent. They therefore represent feedback-centered comparators rather than full reproductions of MAPLe-I's joint diagnosis–policy–outcome architecture.

#### 4.1.4 Learner modeling and academic prediction baselines

Three additional baselines were used to test whether MAPLe-I provides benefits beyond diagnosis or prediction alone:

- **GKTP**, as a graph-based knowledge tracing baseline (Wang et al., 2025);
- **LASA**, as a long-term academic outcome prediction baseline (Ren and Yu, 2024);
- **XGB-SHAP**, as an interpretable academic prediction baseline (Wang and Luo, 2024).

#### 4.1.5 Baseline roles

Table 3 summarizes the baseline methods and their respective roles in the evaluation framework.

**Table 3:** Baseline methods and their roles in the evaluation framework.

Baseline	Category	Role in evaluation
Non-personalized digital intervention	Traditional control	Evaluates whether personalized intervention improves outcomes over uniform support.
AL / CogBooks	Adaptive learning	Evaluates whether MAPLe-I outperforms traditional adaptive learning.
Adaptive Feedback	AI-based feedback	Evaluates whether MAPLe-I outperforms adaptive feedback logic alone.
GenAI Feedback	AI-based feedback	Evaluates whether MAPLe-I outperforms generative-AI-based feedback alone.
Hybrid Feedback	AI-based feedback	Evaluates whether MAPLe-I outperforms combined adaptive and generative feedback.
GKTP	Learner modeling	Evaluates whether MAPLe-I improves learner-state modeling beyond graph-based knowledge tracing.
LASA	Long-term prediction	Evaluates whether MAPLe-I improves long-horizon academic outcome prediction.
XGB-SHAP	Interpretable prediction	Evaluates whether MAPLe-I improves beyond interpretable academic prediction alone.

### 4.2 Evaluation metrics

Because MAPLe-I jointly addresses short-term learning performance, long-term academic development, and intervention quality, multiple evaluation metrics were used.

#### 4.2.1 Short-term learning metrics

For short-term classification-oriented tasks such as next-response correctness, Accuracy, Precision, Recall, F1, and AUC were used:


$$\begin{array}{l} \text{Accuracy}=\frac{TP+TN}{TP+TN+FP+FN}, \end{array}$$ (58)

$$\begin{array}{l} \text{Precision}=\frac{TP}{TP+FP}, \end{array}$$ (59)

$$\begin{array}{l} \text{Recall}=\frac{TP}{TP+FN}, \end{array}$$ (60)

$$\begin{array}{l} \text{F1}=\frac{2\times \text{Precision}\times \text{Recall}}{\text{Precision}+\text{Recall}}, \end{array}$$ (61)

$$\begin{array}{l} \text{AUC}=\int_{0}^{1}TPR\left(FPR^{-1}\left(x\right)\right)dx. \end{array}$$ (62)

Among these, F1 and AUC were treated as the principal short-term metrics because they are more robust than raw accuracy when the class distribution is imbalanced.

#### 4.2.2 Long-term academic outcome metrics

For long-term academic outcomes, Macro-F1 and AUC were used for classification-oriented settings, whereas MAE and RMSE were used for regression-oriented settings:


$$\begin{array}{l} \text{MAE}=\frac{1}{N}\overset{N}{\underset{i=1}{\sum }}|y_{i}-{ŷ}_{i}|, \end{array}$$ (63)

$$\begin{array}{l} \text{RMSE}=\sqrt{\frac{1}{N}\overset{N}{\underset{i=1}{\sum }}{\left(y_{i}-{ŷ}_{i}\right)}^{2}}. \end{array}$$ (64)

#### 4.2.3 Intervention quality metrics

To evaluate intervention-related behavior under the present offline setup, two additional indicators were used.

The first is the **Potential-Improvement Index**:


$$\begin{array}{l} \Delta_{i}={ŷ}_{i}^{\left(long\right)}-y_{i}^{\left(base\right)}, \end{array}$$ (65)

where ${ŷ}_{i}^{\left(long\right)}$ denotes the predicted long-term academic outcome and $y_{i}^{\left(base\right)}$ denotes the learner's baseline or expected outcome level. As noted above, this is a model-based descriptor rather than an independently validated developmental measure.

The second is the **Intervention Alignment Rate (IAR)**:


$$\begin{array}{l} \text{IAR}=\frac{1}{\sum_{i}T_{i}}\overset{N}{\underset{i=1}{\sum }}\overset{T_{i}}{\underset{t=1}{\sum }}I\left(a_{i,t}=a_{i,t}^{target}\right), \end{array}$$ (66)

where $a_{i,t}^{target}$ denotes the held-out rule-consistent pedagogical target generated by the same rule schema, rather than a directly observed teacher intervention. Therefore, IAR quantifies agreement with the predefined pedagogical rule system and should not be interpreted as direct evidence of authentic intervention quality.

In addition, subgroup-specific analyses were conducted for the underachieving, at-risk, and low-potential-gap groups in order to examine whether the proposed framework is especially informative for learners whose current performance does not fully reflect their model-estimated longer-horizon outcomes.

### 4.3 Experimental settings

#### 4.3.1 Dataset allocation

Three public datasets were used in the experiments:

- **ASSISTments 2012–2013 with Affect** for full-model evaluation of learner-state encoding, mechanism estimation, intervention alignment, and short-term learning outcomes;
- **EdNet-KT1** for external validation and generalization testing in a large-scale sequential learning setting;
- **OULAD** for long-term academic outcome evaluation at the course level.

#### 4.3.2 Data preprocessing

Invalid and duplicated records were removed, continuous variables were standardized, categorical variables were embedded, and learner interactions were chronologically ordered. The data were partitioned at the learner level to avoid information leakage, using a 70%/10%/20% split for training, validation, and testing, respectively.

#### 4.3.3 Implementation details

For MAPLe-I, the hidden dimension was set to 128. The graph encoder used two graph propagation layers, and the sequence encoder used a two-layer Transformer with four attention heads. The mechanism head produced three latent mechanism scores corresponding to engagement-related state, self-regulation-related readiness, and affective support need. The model was optimized using Adam with a learning rate of 1 × 10^−3^, weight decay of 1 × 10^−5^, and batch size of 64. Early stopping with a patience of 10 epochs was applied on the validation set.

The loss coefficients in Equation 47 were set as follows:


$$\lambda_{1}=1.0,\;\lambda_{2}=1.0,\;\lambda_{3}=0.3,\;\lambda_{4}=0.5,\;\lambda_{5}=1\times 10^{-5}.$$

The default settings adopted in the main experiments were further validated through the parameter sensitivity analysis reported in Section 4.6.

From an implementation perspective, MAPLe-I involves a deliberate performance–complexity trade-off. The graph encoder and two-layer Transformer improve representation quality, but they also increase memory use and inference overhead relative to rule-only or shallow predictive baselines. In the present setup, intervention generation requires access to recent interaction histories, concept relations, and affect-related proxy features, which is realistic for learning management systems that already log item-level events but may be harder to support in low-infrastructure settings. For multi-user deployment, practical implementations may rely on asynchronous server-side scoring, minibatch inference, or periodic state refresh rather than strict per-click recomputation. When lower latency is prioritized, simplified variants could remove the graph component, reduce the hidden dimension or attention heads, or distill the mechanism layer into a lighter classifier while retaining the same intervention categories.

#### 4.3.4 Baseline implementation

All baselines were implemented under the same learner-level data partitions, preprocessing pipeline, and evaluation protocol. The non-personalized baseline used fixed progression and fixed feedback. The adaptive learning baseline used rule-based progression and mastery updating without a latent proxy layer. The three feedback baselines shared the same learner summaries but differed in whether pedagogical responses were produced by adaptive rules, a generative model, or a hybrid of both. GKTP, LASA, and XGB-SHAP were implemented as diagnosis- or prediction-oriented comparators using the same task-specific inputs available under each dataset. To avoid overstating reproducibility, these baselines should be understood as carefully aligned reference implementations designed for controlled comparison rather than official replications of commercial or previously published systems.

Recent Transformer-based knowledge tracing models such as SAKT, AKT, and SAINT were also considered during benchmark design because they are important state-of-the-art references for sequential learner modeling. They were not included in the main empirical tables for two methodological reasons. First, the present benchmark jointly evaluates short-term prediction, intervention alignment, and longer-horizon academic outcome modeling across three datasets, whereas these KT models are typically formulated for next-response prediction and do not natively output pedagogical actions or course-level outcome estimates under a unified protocol. Second, a faithful comparison would require substantial task-specific re-engineering beyond their original design assumptions, which could introduce implementation asymmetries that are difficult to interpret fairly. For this reason, the manuscript now discusses them explicitly in the related-work positioning while retaining the present baseline set for controlled cross-task comparison.

#### 4.3.5 Statistical significance testing

To verify that the observed gains of MAPLe-I were not caused by random variation, statistical significance testing was conducted using the raw metric values obtained from five independent runs with different random seeds. Specifically, all methods were trained and evaluated under the same data partitions and experimental settings, and the random seeds were fixed across methods to ensure fair comparison. For each comparison, the reported *p*-values were computed from the seed-level metric vectors rather than from the summary mean values reported in the main result tables.

Formally, let ${\text{s}}^{\left(M\right)}=\left\{s_{1}^{\left(M\right)},s_{2}^{\left(M\right)},\ldots ,s_{5}^{\left(M\right)}\right\}$ denote the metric values of MAPLe-I over five runs, and let ${\text{s}}^{\left(B\right)}=\left\{s_{1}^{\left(B\right)},s_{2}^{\left(B\right)},\ldots ,s_{5}^{\left(B\right)}\right\}$ denote the corresponding values of the compared baseline. For each pairwise comparison, the difference sequence was defined as


$$\begin{array}{l} d_{j}=s_{j}^{\left(M\right)}-s_{j}^{\left(B\right)},\;j=1,2,\ldots ,5. \end{array}$$ (67)

A paired *t*-test was then performed on {*d*_1_, *d*_2_, …, *d*_5_} to determine whether the mean difference was significantly different from zero. For metrics in which lower values indicate better performance, such as RMSE and MAE, the same paired testing procedure was applied directly to the raw metric values.

### 4.4 Main results

#### 4.4.1 Short-term learning performance on ASSISTments

Table 4 reports the short-term learning performance on ASSISTments 2012–2013 with Affect. Overall, a clear performance hierarchy can be observed. The non-personalized digital intervention baseline produced the weakest results, suggesting that uniform digital support is insufficient for heterogeneous learners. The traditional adaptive learning baseline improved substantially over the non-personalized condition, indicating that even rule-based personalization can produce measurable benefits. The three AI-based feedback baselines further improved performance, with Hybrid Feedback outperforming both Adaptive Feedback and GenAI Feedback, suggesting that combining adaptive logic with generative feedback is more effective than relying on either feedback source alone.

**Table 4:** Short-term performance on ASSISTments 2012–2013 with affect (mean ± std, five seeds).

Method	Acc.	F1	AUC	IAR
Non-personalized Interv.	0.652 ± 0.005	0.648 ± 0.006	0.675 ± 0.005	0.534 ± 0.008
AL / CogBooks	0.684 ± 0.004	0.680 ± 0.005	0.712 ± 0.004	0.585 ± 0.007
Adaptive FB	0.705 ± 0.006	0.712 ± 0.005	0.738 ± 0.006	0.642 ± 0.006
GenAI FB	0.718 ± 0.005	0.725 ± 0.006	0.751 ± 0.005	0.668 ± 0.005
Hybrid FB	0.735 ± 0.004	0.740 ± 0.005	0.768 ± 0.004	0.705 ± 0.004
GKTP	0.752 ± 0.003	0.756 ± 0.004	0.785 ± 0.003	N/A
LASA	0.728 ± 0.004	0.732 ± 0.003	0.755 ± 0.005	N/A
XGB-SHAP	0.715 ± 0.005	0.720 ± 0.004	0.742 ± 0.006	N/A
**MAPLe-I**	**0.786** **±** **0.002**	**0.792** **±** **0.003**	**0.824 ± 0.002** ^**^	**0.785 ± 0.003** ^**^

Higher is better.

Underlined values indicate the second-best result under the same metric.

Bold values indicate the best result under the same metric.

Among the learner modeling and academic prediction baselines, GKTP performed most strongly in Accuracy, F1, and AUC, indicating that high-quality learner-state modeling contributes substantially to short-term learning prediction. However, MAPLe-I achieved the best overall results across all reported metrics. Compared with the strongest baseline on AUC, namely GKTP, MAPLe-I improved AUC from 0.785 to 0.824. Compared with the strongest intervention-oriented baseline on IAR, namely Hybrid Feedback, MAPLe-I improved IAR from 0.705 to 0.785. These results indicate that the proposed framework not only provides stronger short-term prediction, but also generates support actions that are more closely aligned with the held-out rule-consistent pedagogical targets used in this benchmark.

**Figure 2:**
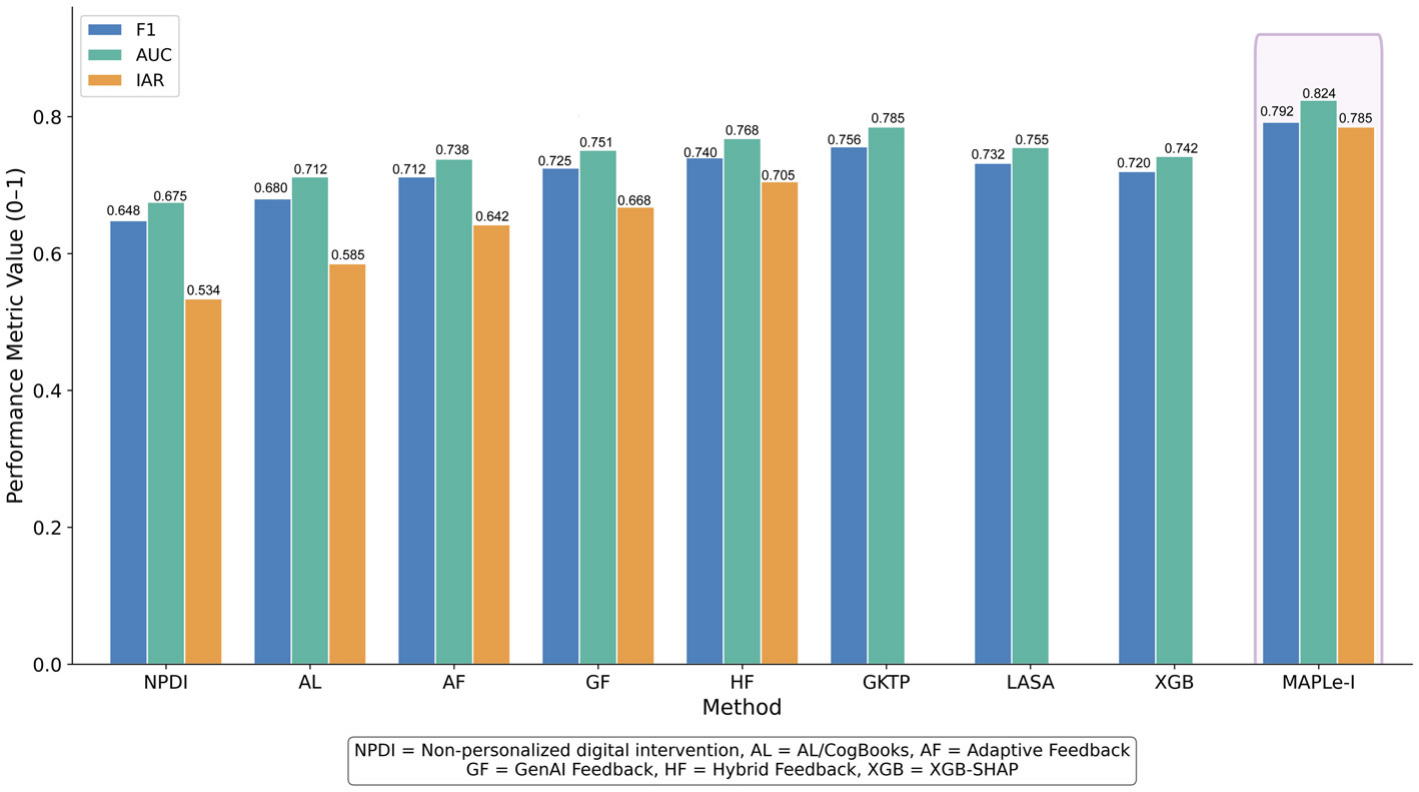
Comparison of short-term learning performance on ASSISTments 2012–2013 with Affect. The figure visualizes the comparative results of all methods in terms of F1, AUC, and Intervention Alignment Rate (IAR). MAPLe-I achieved the strongest overall performance, indicating superior short-term learner-state modeling and stronger agreement between generated support actions and the held-out rule-consistent pedagogical targets used in this offline benchmark.

The comparative pattern can be more clearly observed in Figure 2, where MAPLe-I consistently outperforms the baseline methods in terms of F1, AUC, and Intervention Alignment Rate. Taken together, Table 4 and Figure 2 indicate that MAPLe-I provides both stronger learner-state prediction and stronger agreement with the held-out rule-based support targets on ASSISTments.

Importantly, these gains should be interpreted as stronger predictive and simulated policy-alignment performance under an offline benchmark. In this study, higher IAR indicates closer agreement with held-out rule-consistent pedagogical targets, not verified superiority of authentic classroom interventions.

#### 4.4.2 Generalization performance on EdNet-KT1

Table 5 reports the large-scale generalization performance on EdNet-KT1. Overall, the performance pattern remained broadly consistent with that observed on ASSISTments, but the learner-modeling baseline GKTP became more competitive in this setting. This result is theoretically reasonable because EdNet-KT1 is a larger and more sequentially structured dataset, making strong knowledge tracing methods particularly effective.

**Table 5:** Comparison of large-scale generalization performance on EdNet-KT1 (mean ± standard deviation over five random seeds).

Method	Accuracy	F1	AUC
Non-personalized digital intervention	0.665 ± 0.005	0.660 ± 0.004	0.692 ± 0.005
AL / CogBooks	0.685 ± 0.004	0.682 ± 0.005	0.715 ± 0.004
Adaptive Feedback	0.702 ± 0.005	0.700 ± 0.006	0.732 ± 0.005
GenAI Feedback	0.715 ± 0.004	0.712 ± 0.005	0.745 ± 0.004
Hybrid Feedback	0.728 ± 0.003	0.725 ± 0.004	0.758 ± 0.003
GKTP	0.745 ± 0.004	0.748 ± 0.003	0.778 ± 0.004
LASA	0.720 ± 0.005	0.722 ± 0.004	0.750 ± 0.005
XGB-SHAP	0.710 ± 0.004	0.715 ± 0.005	0.740 ± 0.006
**MAPLe-I**	**0.775** **±** **0.002**	**0.780** **±** **0.003**	**0.812 ± 0.003** ^**^

Higher values indicate better performance.

Underline indicates the second-best result under the same metric.

Bold values indicate the best result under the same metric.

^**^ indicates that MAPLe-I significantly outperformed the strongest corresponding baseline under the same metric at *p* < 0.01.

Among the compared baselines, GKTP achieved the best Accuracy, F1, and AUC, indicating that graph-based learner-state modeling provides a strong benchmark in large-scale interaction environments. Nevertheless, MAPLe-I achieved the best overall performance across all three metrics, reaching 0.775 in Accuracy, 0.780 in F1, and 0.812 in AUC. Compared with GKTP, MAPLe-I improved Accuracy by 0.030, F1 by 0.032, and AUC by 0.034. These results suggest that the superiority of the proposed framework is not restricted to the primary dataset, but generalizes to a larger and more behaviorally diverse educational environment.

**Figure 3:**
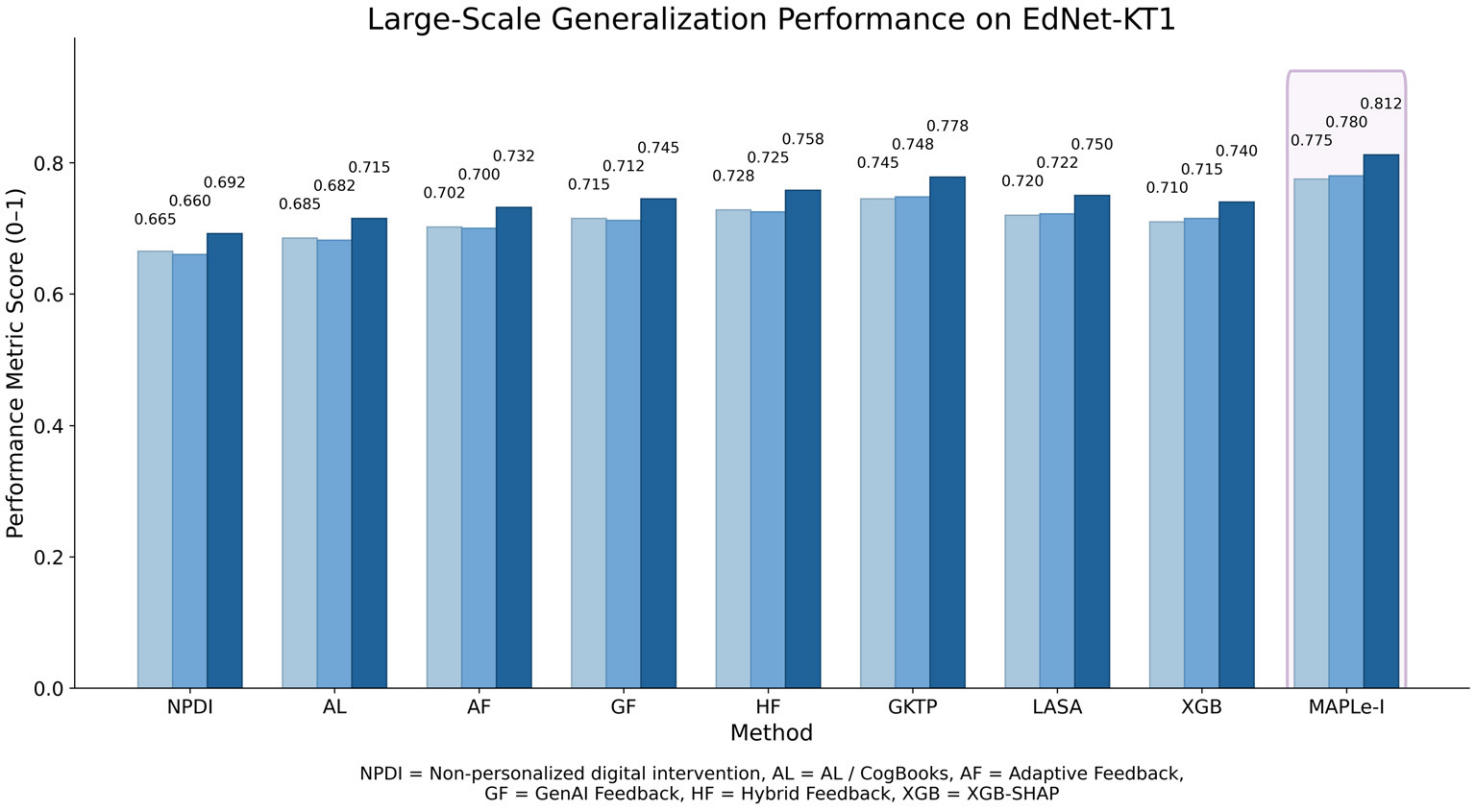
Large-scale generalization performance on EdNet-KT1. The figure presents the comparative Accuracy, F1, and AUC values of all methods under a large-scale external validation setting. MAPLe-I consistently outperformed the strongest baseline methods, suggesting that the proposed framework generalizes effectively to larger and more behaviorally diverse sequential learning environments.

Figure 3 further visualizes the generalization performance on EdNet-KT1, showing that MAPLe-I maintains clear advantages over the strongest baseline methods in a larger and more behaviorally diverse learning environment. The consistency between Table 5 and Figure 3 suggests that the performance gains of MAPLe-I are robust under large-scale sequential learning conditions.

#### 4.4.3 Long-term academic performance on OULAD

Table 6 reports the long-term academic performance on OULAD. Overall, the results show a clear progression from non-personalized digital intervention to adaptive learning, AI-based feedback methods, learner modeling baselines, and finally the proposed MAPLe-I framework. The non-personalized baseline yielded the weakest results across all metrics, whereas adaptive learning and feedback-based baselines showed consistent improvements, indicating that personalized support is beneficial for long-term academic development.

**Table 6:** Long-term performance on OULAD (mean ± std, five seeds).

Method	M-F1 ↑	AUC ↑	RMSE ↓	MAE ↓
Non-personalized Interv.	0.650 ± 0.007	0.685 ± 0.006	0.455 ± 0.008	0.375 ± 0.008
AL / CogBooks	0.675 ± 0.006	0.710 ± 0.004	0.420 ± 0.007	0.345 ± 0.006
Adaptive FB	0.688 ± 0.005	0.725 ± 0.005	0.405 ± 0.006	0.328 ± 0.005
GenAI FB	0.695 ± 0.004	0.732 ± 0.006	0.395 ± 0.005	0.320 ± 0.004
Hybrid FB	0.705 ± 0.005	0.745 ± 0.005	0.385 ± 0.004	0.312 ± 0.005
GKTP	0.715 ± 0.006	0.752 ± 0.004	0.380 ± 0.005	0.305 ± 0.006
LASA	0.735 ± 0.005	0.768 ± 0.004	0.358 ± 0.005	0.291 ± 0.004
XGB-SHAP	0.728 ± 0.004	0.760 ± 0.005	0.365 ± 0.006	0.285 ± 0.005
**MAPLe-I**	**0.772 ± 0.004** ^**^	**0.805 ± 0.003** ^**^	**0.315 ± 0.004** ^**^	**0.242 ± 0.003** ^**^

Higher is better for Macro-F1 and AUC; lower is better for RMSE and MAE.

Underline: second-best result.

Bold values indicate the best result under the same metric.

^**^: significantly better than the strongest baseline under the same metric (*p* < 0.01).

Among the stronger prediction-oriented baselines, LASA achieved the best Macro-F1, AUC, and RMSE, while XGB-SHAP obtained the lowest MAE among the compared baselines. This pattern is reasonable because LASA was specifically designed for long-term student performance prediction, whereas XGB-SHAP provides a strong interpretable machine-learning benchmark for structured educational data. Nevertheless, MAPLe-I achieved the best overall performance across all four metrics, reaching 0.772 in Macro-F1, 0.805 in AUC, 0.315 in RMSE, and 0.242 in MAE.

Compared with LASA, MAPLe-I improved Macro-F1 by 0.037 and AUC by 0.037, while reducing RMSE by 0.043. Compared with XGB-SHAP, MAPLe-I reduced MAE by 0.043. These results suggest that the proposed framework contributes not only to immediate prediction quality, but also to longer-term academic outcome modeling under the present offline design. From a methodological perspective, the findings indicate that combining learner-state diagnosis, latent proxy estimation, personalized intervention policy learning, and dual-horizon optimization provides advantages beyond long-term prediction or interpretable modeling alone in benchmark settings.

Because these OULAD results are still obtained under an offline predictive setup, they should be read as evidence of stronger long-horizon academic outcome modeling rather than direct proof that the recommended interventions would improve real course outcomes if deployed unchanged.

**Figure 4:**
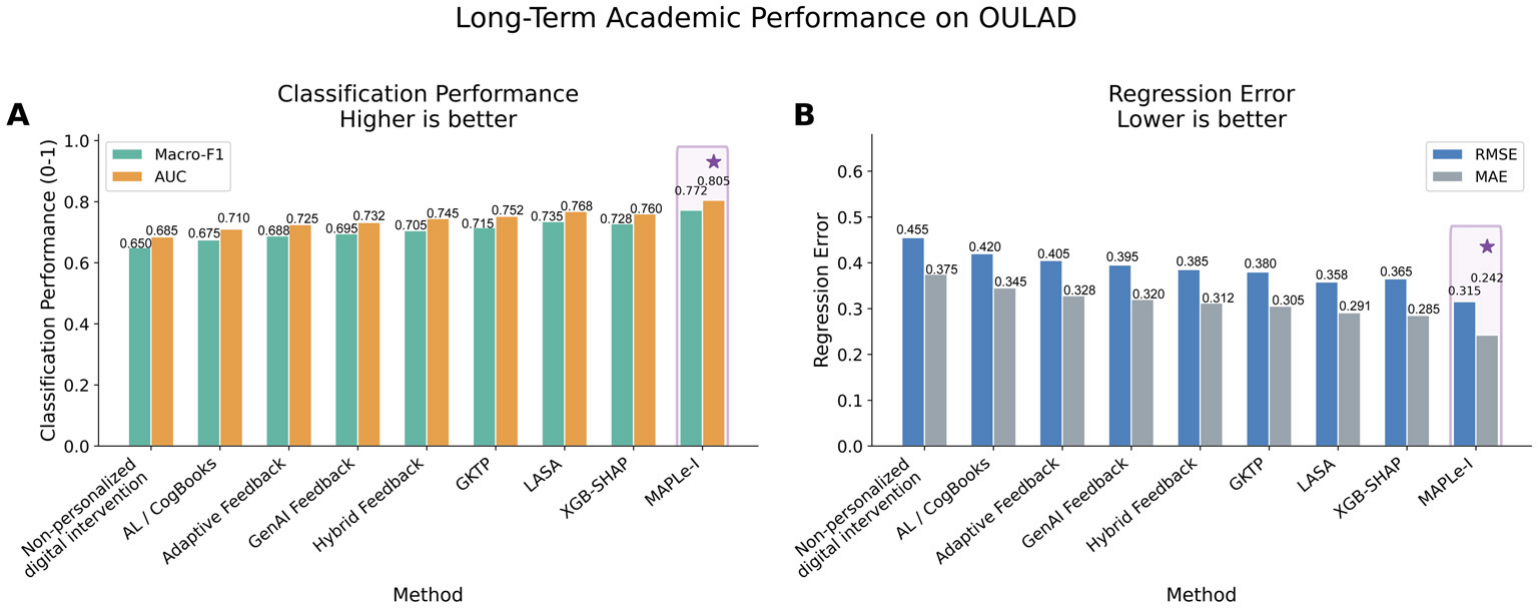
Long-term academic performance on OULAD. The figure summarizes the comparative results of all methods in terms of Macro-F1, AUC, RMSE, and MAE. MAPLe-I achieved the best overall performance, indicating that the proposed framework improves not only immediate predictive quality but also longer-term academic outcome modeling.

Figure 4 provides a more intuitive view of the long-term performance differences, highlighting that MAPLe-I improves Macro-F1 and AUC while simultaneously reducing RMSE and MAE. Together, Table 6 and Figure 4 support the conclusion that MAPLe-I contributes not only to immediate predictive effectiveness but also to broader academic development over time.

#### 4.4.4 Potential-improvement analysis

To further evaluate the developmental benefit of the proposed framework, the Potential-Improvement Index was computed across datasets relative to the non-personalized digital intervention baseline. The results consistently showed that MAPLe-I achieved the largest gains among all compared methods, indicating that the proposed framework is particularly effective in supporting learners whose observed performance may underestimate their developmental potential. This pattern is theoretically consistent with the mechanism-aware and dual-horizon design of the model.

#### 4.4.5 Statistical significance analysis

To further verify the robustness of the observed gains, paired statistical significance tests were conducted between MAPLe-I and the strongest corresponding baselines, as shown in Table 7. The results indicate that the advantages of MAPLe-I were statistically significant across short-term learning performance, intervention alignment, cross-dataset generalization, and long-term academic outcome prediction.

**Table 7:** Statistical significance tests between MAPLe-I and the strongest corresponding baselines (mean ± standard deviation over five random seeds).

Comparison	Metric	MAPLe-I	Baseline	*p*-value
MAPLe-I vs. GKTP (ASSISTments)	AUC	0.824 ± 0.002	0.785 ± 0.003	< 0.001
MAPLe-I vs. Hybrid Feedback (ASSISTments)	IAR	0.785 ± 0.003	0.705 ± 0.004	< 0.001
MAPLe-I vs. GKTP (EdNet-KT1)	AUC	0.812 ± 0.003	0.778 ± 0.004	0.002
MAPLe-I vs. LASA (OULAD)	Macro-F1	0.772 ± 0.004	0.735 ± 0.005	< 0.001
MAPLe-I vs. LASA (OULAD)	AUC	0.805 ± 0.003	0.768 ± 0.004	< 0.001
MAPLe-I vs. LASA (OULAD)	RMSE	0.315 ± 0.004	0.358 ± 0.005	< 0.001
MAPLe-I vs. XGB-SHAP (OULAD)	MAE	0.242 ± 0.003	0.285 ± 0.005	0.004

The reported *p*-values were obtained from paired *t*-tests based on the raw metric values from five runs with different random seeds.

For AUC, IAR, and Macro-F1, higher values indicate better performance. For RMSE and MAE, lower values indicate better performance.

On ASSISTments 2012–2013 with Affect, MAPLe-I significantly outperformed GKTP in terms of AUC and significantly outperformed Hybrid Feedback in terms of IAR, indicating stronger short-term learner-state modeling and more effective intervention alignment than the strongest learner-modeling and intervention-oriented baselines, respectively. On EdNet-KT1, MAPLe-I again achieved a significantly higher AUC than GKTP, suggesting that the superiority of the proposed framework generalized to a larger and more behaviorally diverse learning environment.

On OULAD, MAPLe-I significantly outperformed LASA in terms of Macro-F1, AUC, and RMSE, and significantly outperformed XGB-SHAP in terms of MAE. These findings indicate that the benefits of MAPLe-I extend beyond short-term learning prediction to longer-term academic outcome modeling. Taken together, the significance results strengthen the central claim of this study: the superiority of MAPLe-I is not attributable to random variation across runs, but reflects a consistent and statistically reliable improvement over the strongest corresponding baselines across multiple datasets and evaluation dimensions.

**Figure 5:**
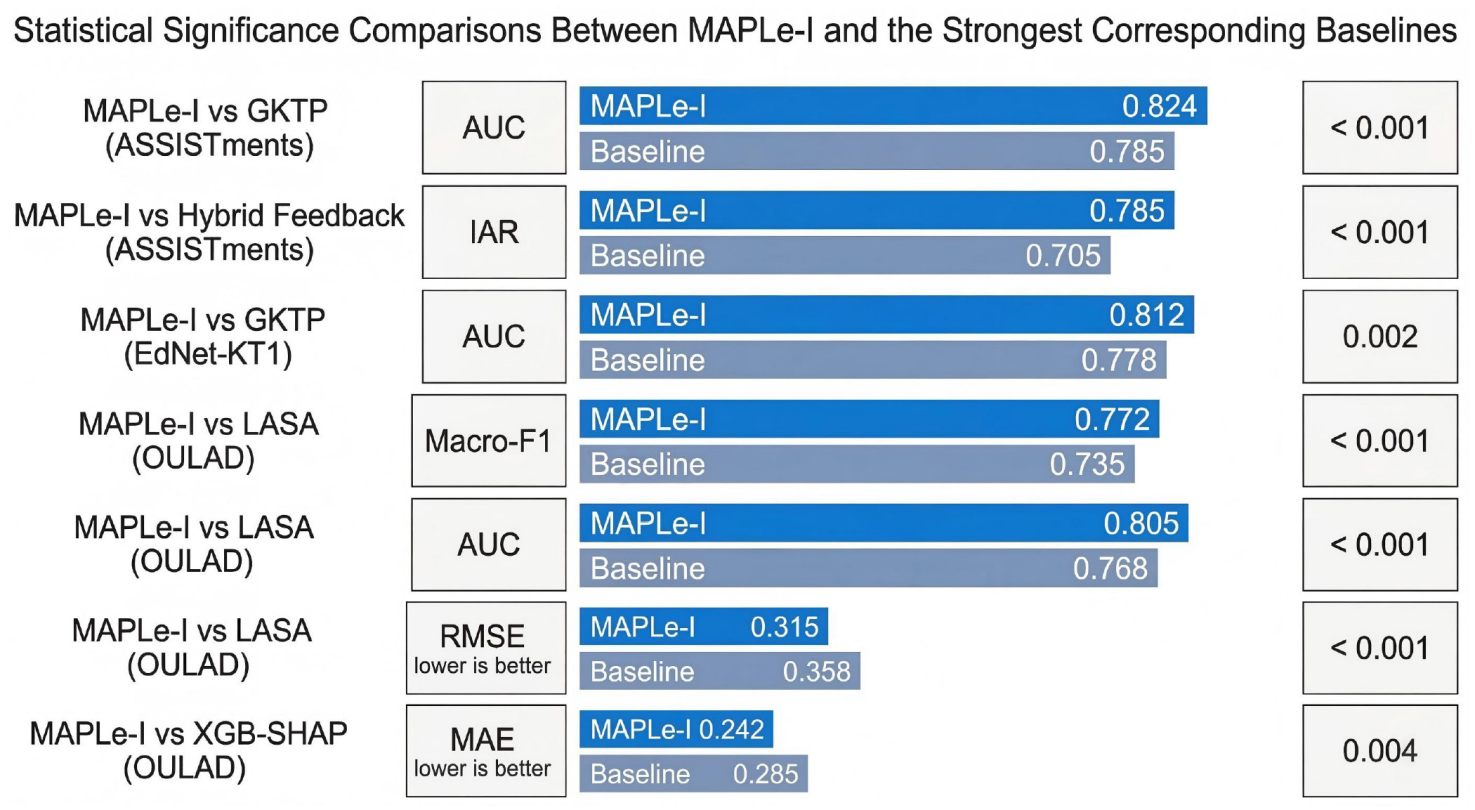
Statistical significance comparisons between MAPLe-I and the strongest corresponding baselines. The figure summarizes the pairwise comparisons under the key evaluation metrics used in this study, together with the associated *p*-values. The results show that the performance advantages of MAPLe-I are statistically significant across short-term learning performance, intervention alignment, cross-dataset generalization, and long-term academic outcome prediction.

Figure 5 complements Table 7 by providing a visual summary of the pairwise significance comparisons between MAPLe-I and the strongest corresponding baselines. The statistical evidence reported in Table 7 and Figure 5 confirms that the superiority of MAPLe-I is not attributable to random variation across runs.

### 4.5 Ablation study

To further examine the contribution of each component in MAPLe-I, an ablation study was conducted by progressively removing key modules of the framework, as shown in Table 8. Overall, the complete MAPLe-I model achieved the best performance on all three datasets, indicating that the proposed architecture benefits from the joint integration of learner-state encoding, mechanism estimation, intervention policy learning, and dual-horizon outcome optimization.

**Table 8:** Ablation results of MAPLe-I on ASSISTments, EdNet-KT1, and OULAD (mean ± standard deviation over five random seeds).

Variant	ASSISTments AUC	EdNet-KT1 AUC	OULAD Macro-F1
w/o Mechanism Head	0.792 ± 0.004	0.785 ± 0.004	0.735 ± 0.005
w/o Policy Head	0.801 ± 0.003	0.792 ± 0.003	0.742 ± 0.006
w/o Dual Outcome Optimization	0.812 ± 0.003	0.804 ± 0.004	0.715 ± 0.005
w/o Graph Encoder	0.808 ± 0.004	0.798 ± 0.005	0.755 ± 0.004
w/o Temporal Encoder	0.785 ± 0.005	0.775 ± 0.006	0.730 ± 0.006
**MAPLe-I**	**0.824** **±** **0.002**	**0.812** **±** **0.003**	**0.772** **±** **0.004**

Higher values indicate better performance.

Underline indicates the second-best result under the same metric.

Bold values indicate the best result under the same metric.

Removing the mechanism estimation head led to a clear decline across all datasets, suggesting that the mechanism-aware component contributes substantially to both short-term and long-term prediction by refining learner-state representation with intervention-relevant latent information. Removing the intervention policy head also resulted in consistent degradation, indicating that accurate learner-state diagnosis alone is insufficient unless it is translated into personalized support actions.

Interestingly, removing the temporal encoder produced the largest decline on the two sequential short-term datasets, namely ASSISTments and EdNet-KT1. This finding suggests that temporal dependency modeling forms the structural backbone of learner-state diagnosis in interaction-rich environments. By contrast, removing dual outcome optimization caused the largest degradation on OULAD, where the Macro-F1 value dropped most severely. This result is theoretically consistent with the design of the long-term outcome head, indicating that dual-horizon optimization is particularly important for broader academic development rather than only immediate performance prediction.

Taken together, the ablation results suggest that different modules contribute in complementary ways: the temporal encoder provides the foundational sequential learner-state representation, the mechanism estimation head adds intervention-relevant refinement, the policy head converts diagnosis into action, and dual outcome optimization strengthens long-term academic modeling. These findings suggest the central claim of this study that the effectiveness of MAPLe-I arises from the coordinated interaction of its components rather than from any single module alone.

**Figure 6:**
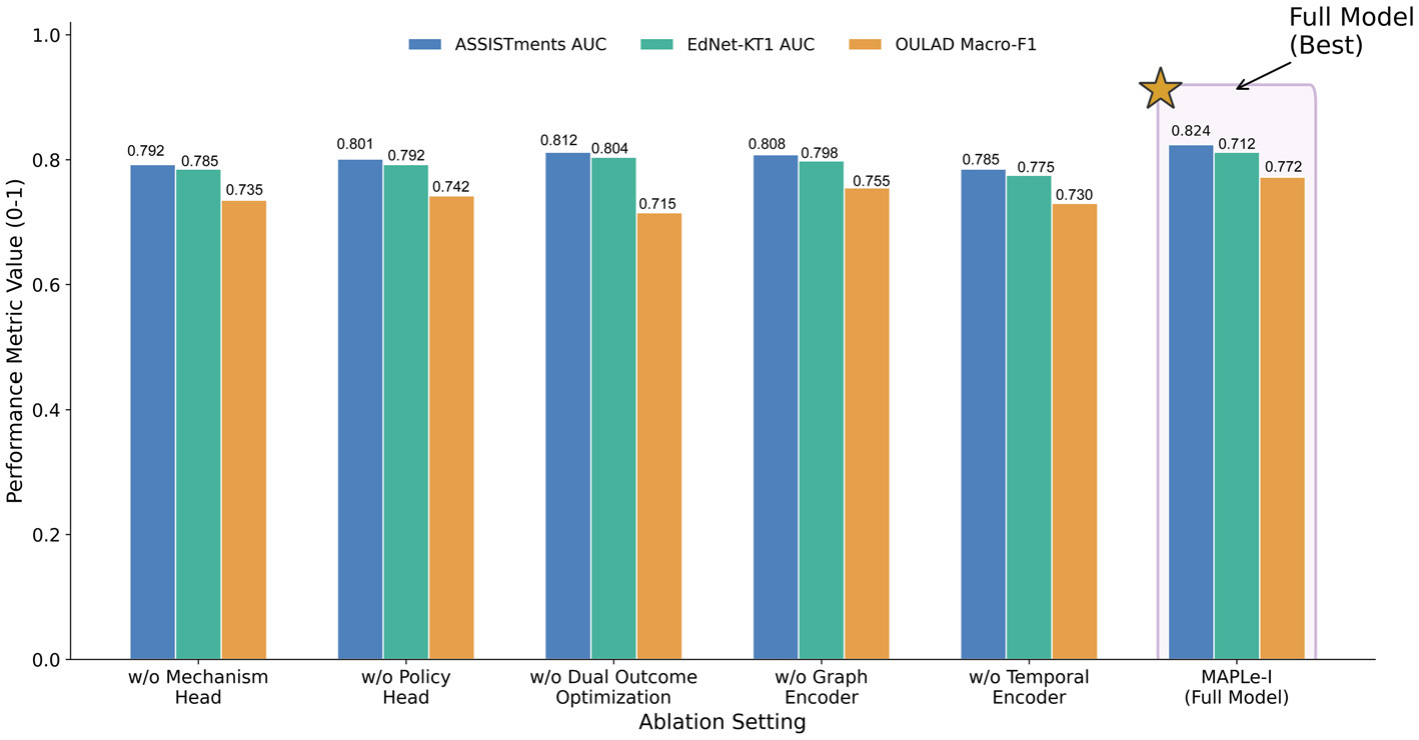
Ablation analysis of MAPLe-I across ASSISTments, EdNet-KT1, and OULAD. The figure compares the complete model with several ablated variants, including removal of the mechanism estimation head, intervention policy head, dual outcome optimization, graph encoder, and temporal encoder. The full MAPLe-I model consistently achieved the best performance, confirming that the effectiveness of the framework arises from the coordinated contribution of its components.

Figure 6 provides a visual comparison between the complete MAPLe-I model and its ablated variants across the three datasets. Taken together, Table 8 and Figure 6 suggest that the effectiveness of MAPLe-I arises from the coordinated interaction of learner-state encoding, mechanism estimation, intervention policy learning, and dual-horizon optimization.

### 4.6 Parameter sensitivity analysis

**Figure 7:**
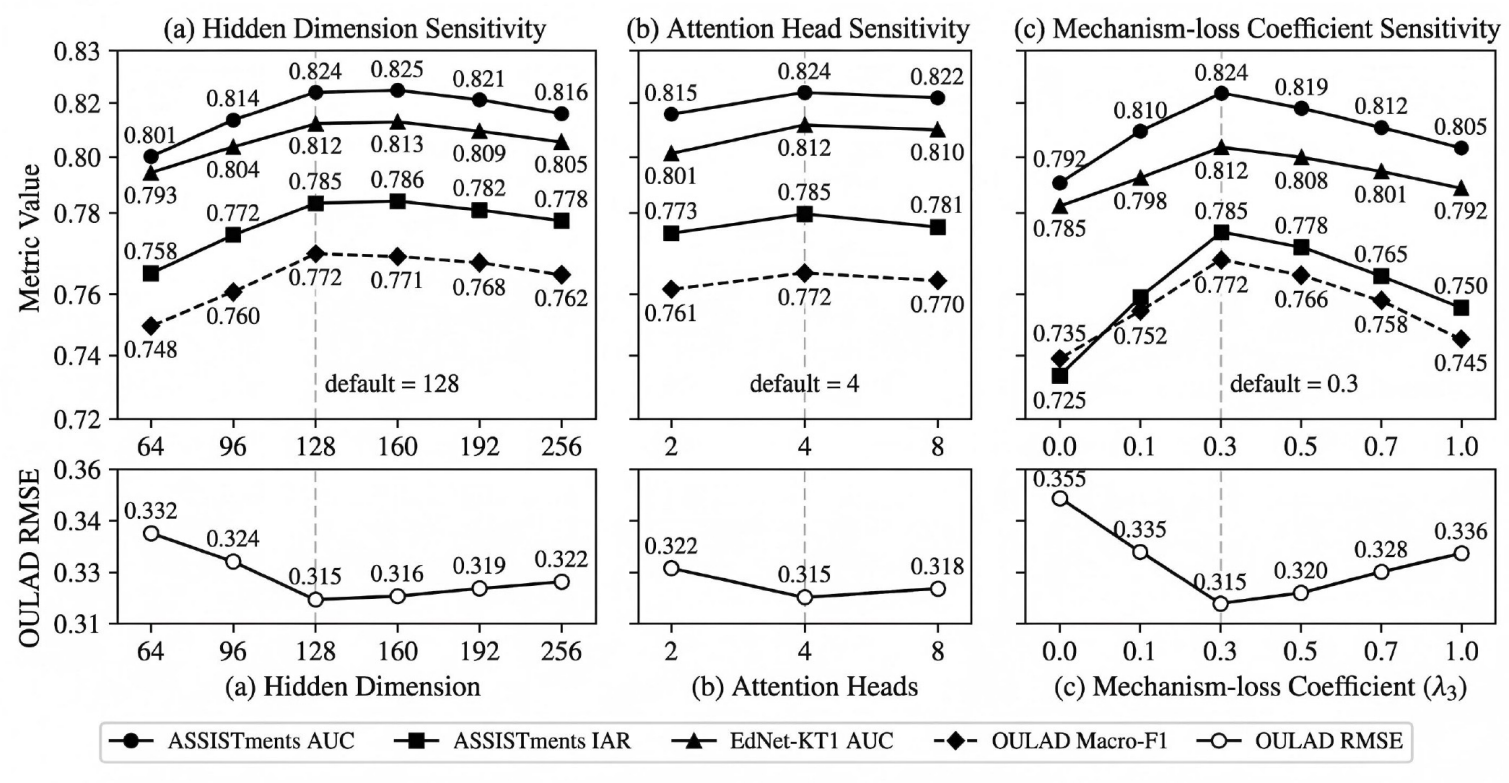
Parameter sensitivity analysis of MAPLe-I with respect to hidden dimension size, number of Transformer attention heads, and mechanism-loss coefficient λ_3_. **(a)** shows the effect of hidden dimension size, **(b)** shows the effect of attention heads, and **(c)** shows the effect of the mechanism-loss coefficient. In each panel, the upper subfigure reports the higher-is-better metrics (ASSISTments AUC, ASSISTments IAR, EdNet-KT1 AUC, and OULAD Macro-F1), while the lower subfigure reports OULAD RMSE separately. Overall, MAPLe-I remains stable under moderate parameter variation, and the default configuration (hidden dimension = 128, attention heads = 4, and λ_3_ = 0.3) provides the best overall trade-off across short-term prediction, intervention alignment, cross-dataset generalization, and long-term academic outcome modeling.

To assess the robustness of MAPLe-I with respect to key hyperparameters, a parameter sensitivity analysis was conducted on the hidden dimension size, the number of Transformer attention heads, and the mechanism-loss coefficient λ_3_. Following the implementation settings described in Section 4.3.3, the default configuration used a hidden dimension of 128, a two-layer Transformer with 4 attention heads, Adam optimization with a learning rate of 1 × 10^−3^, weight decay of 1 × 10^−5^, a batch size of 64, and early stopping on the validation set with a patience of 10 epochs. Hyperparameter selection was guided by validation-set performance, whereas the final results reported in Tables 9–11 and Figure 7 were obtained by evaluating the best checkpoint of each setting on the test set. Each setting was repeated over five random seeds, and the mean ± standard deviation is reported. To avoid redundancy, only the primary task-specific metrics are shown, namely AUC and IAR for ASSISTments, AUC for EdNet-KT1, and Macro-F1 together with RMSE for OULAD.

**Table 9:** Parameter sensitivity analysis of hidden dimension size (mean ± standard deviation over five random seeds).

Hidden dimension	ASSISTments (AUC)	ASSISTments (IAR)	EdNet-KT1 (AUC)	OULAD (macro-F1)	OULAD (RMSE)
64	0.801 ± 0.003	0.758 ± 0.004	0.793 ± 0.004	0.748 ± 0.005	0.332 ± 0.004
96	0.814 ± 0.004	0.772 ± 0.003	0.804 ± 0.004	0.760 ± 0.004	0.324 ± 0.005
**128 (Default)**	**0.824** **±** **0.002**	**0.785** **±** **0.003**	**0.812** **±** **0.003**	**0.772** **±** **0.004**	**0.315** **±** **0.004**
160	0.825 ± 0.003	0.786 ± 0.003	0.813 ± 0.002	0.771 ± 0.005	0.316 ± 0.005
192	0.821 ± 0.004	0.782 ± 0.004	0.809 ± 0.004	0.768 ± 0.004	0.319 ± 0.004
256	0.816 ± 0.004	0.778 ± 0.005	0.805 ± 0.004	0.762 ± 0.005	0.322 ± 0.005

Bold values indicate the best result under the same metric.

**Table 10:** Parameter sensitivity analysis of Transformer attention heads (mean ± standard deviation over five random seeds).

Attention heads	ASSISTments (AUC)	ASSISTments (IAR)	EdNet-KT1 (AUC)	OULAD (Macro-F1)	OULAD (RMSE)
2	0.815 ± 0.004	0.773 ± 0.004	0.801 ± 0.004	0.761 ± 0.005	0.322 ± 0.004
**4 (Default)**	**0.824** **±** **0.002**	**0.785** **±** **0.003**	**0.812** **±** **0.003**	**0.772** **±** **0.004**	**0.315** **±** **0.004**
8	0.822 ± 0.003	0.781 ± 0.004	0.810 ± 0.004	0.770 ± 0.005	0.318 ± 0.005

Bold values indicate the best result under the same metric.

**Table 11:** Parameter sensitivity analysis of mechanism-loss coefficient λ_3_ (mean ± standard deviation over five random seeds).

λ_3_ Coefficient	ASSISTments (AUC)	ASSISTments (IAR)	EdNet-KT1 (AUC)	OULAD (Macro-F1)	OULAD (RMSE)
0.0 (No explicit mechanism loss)	0.792 ± 0.004	0.725 ± 0.005	0.785 ± 0.004	0.735 ± 0.005	0.355 ± 0.005
0.1	0.810 ± 0.004	0.755 ± 0.004	0.798 ± 0.004	0.752 ± 0.005	0.335 ± 0.004
**0.3 (Default)**	**0.824** **±** **0.002**	**0.785** **±** **0.003**	**0.812** **±** **0.003**	**0.772** **±** **0.004**	**0.315** **±** **0.004**
0.5	0.819 ± 0.003	0.778 ± 0.004	0.808 ± 0.004	0.766 ± 0.004	0.320 ± 0.005
0.7	0.812 ± 0.004	0.765 ± 0.005	0.801 ± 0.004	0.758 ± 0.005	0.328 ± 0.004
1.0	0.805 ± 0.005	0.750 ± 0.005	0.792 ± 0.005	0.745 ± 0.006	0.336 ± 0.005

Bold values indicate the best result under the same metric.

Tables 9, 10 summarize the performance variations across different hidden dimensions ({64, 96, 128, 160, 192, 256}) and attention-head settings ({2, 4, 8}). Overall, MAPLe-I remained relatively stable across moderate architectural changes, indicating that the proposed framework is not highly sensitive to minor modifications of representational capacity or multi-head attention structure. In particular, the model achieved the best overall cross-dataset trade-off at a hidden dimension of 128. Although a hidden dimension of 160 yielded marginal gains on several short-term metrics, the default setting of 128 provided slightly better long-term performance on OULAD and was therefore retained as the main configuration. By contrast, reducing the hidden dimension to 64 led to a clear drop in performance across all datasets, suggesting that overly restricted representational capacity weakens the model's ability to encode learner state, mechanism information, and intervention-relevant context.

Table 11 presents the impact of the mechanism-loss coefficient λ_3_ over the range {0.0, 0.1, 0.3, 0.5, 0.7, 1.0}. The best overall performance was obtained at the moderate setting λ_3_ = 0.3, which is consistent with the default configuration in Section 4.3.3. When λ_3_ = 0.0, the explicit mechanism-supervision term was removed, resulting in a marked degradation in both predictive and intervention-alignment performance. This degradation pattern is consistent with the importance of mechanism-aware supervision, although it is not strictly equivalent to removing the mechanism head entirely, because the mechanism branch can still influence downstream predictions through the shared forward architecture. As λ_3_ increased beyond the moderate range, performance gradually declined, especially when λ_3_ = 1.0, indicating that excessive emphasis on mechanism estimation can overshadow the optimization of short-term and long-term academic objectives. Taken together, these results suggest that mechanism estimation is most effective when incorporated as a complementary rather than dominant objective.

Figure 7 provides a more intuitive visualization of the trends reported in Tables 9–11. The curves confirm that MAPLe-I is robust under moderate variations of core hyperparameters and that its strongest performance is achieved when representational capacity, attention structure, and mechanism supervision are kept in balance. This finding further supports the stability of the proposed framework and suggests that its empirical gains do not depend on narrow or fragile parameter tuning.

### 4.7 Explanation analysis

To improve interpretability and illustrate how the estimated latent proxy variables influence the intervention decisions of MAPLe-I, a case-level explanation analysis was conducted on representative learners from the ASSISTments dataset. The purpose of this analysis was not to validate construct validity or authentic intervention usefulness, but to examine whether the policy component responded only to recent observable performance or whether it also differentiated intervention profiles according to the latent learner conditions estimated by the mechanism head. This goal is directly aligned with the design of MAPLe-I, in which intervention decisions are generated from a joint representation of learner state and three mechanism dimensions, namely engagement-related state, self-regulation-related readiness, and affective support need.

For interpretive clarity, representative learner profiles were selected to reflect different combinations of recent correctness and the three latent mechanism variables. Table 12 summarizes these cases together with the corresponding intervention profile generated by the MAPLe-I policy head across the five pedagogical action dimensions: feedback selection, hint allocation, difficulty adjustment, remedial support, and pacing control.

**Table 12:** Case-level explanation analysis of MAPLe-I based on representative learner mechanism profiles.

Profile	Recent correctness	Engagement	SRL readiness	Affective need	Feedback	Hint	Difficulty	Remedial	Pacing
Learner A	High	High	High	Low	Affirming	None	Increase	None	Accelerate
Learner B	Moderate	High	Moderate	High	Supportive	Light	Maintain	None	Maintain
Learner C	Low	Low	Low	High	Supportive	Stepwise	Decrease	Targeted practice	Slow
Learner D	High	Low	Moderate	Moderate	Supportive	None	Maintain	None	Maintain

As shown in Table 12, the intervention profiles are broadly consistent with pedagogical expectations derived from the mechanism-aware design of the model. Learner A exhibits high recent correctness together with high engagement and high self-regulation-related readiness, while affective support need remains low. Under this profile, the model does not continue to provide unnecessary scaffolding, but instead selects an intervention pattern characterized by affirming feedback, difficulty increase, and accelerated pacing. This suggests that the policy component identifies readiness for further challenge rather than merely preserving the current level of support.

Learner B and Learner C illustrate a different pattern. Learner B shows moderate recent correctness but remains behaviorally engaged, while affective support need is relatively high and self-regulation-related readiness is not yet strong. In this case, the model produces a support-oriented intervention profile, combining supportive feedback with light hints while maintaining difficulty and pacing. By contrast, Learner C exhibits low recent correctness together with low engagement, low self-regulation-related readiness, and high affective support need. For this more fragile learner condition, the policy shifts toward stronger scaffolding, including supportive feedback, stepwise hints, difficulty reduction, targeted remedial practice, and slower pacing. This contrast suggests that MAPLe-I does not treat all struggling learners as equivalent, but differentiates between temporary difficulty and broader vulnerability in engagement and self-regulation.

Learner D provides a particularly informative contrast. Although recent correctness remains high, the estimated mechanism profile indicates reduced engagement and moderate affective support need. Instead of automatically escalating difficulty, the model retains a more conservative intervention profile, characterized by supportive feedback and maintained pacing. This case suggests that recent correctness alone does not deterministically drive challenge escalation. Rather, the intervention policy appears to weigh whether the learner is in a condition to sustain productive participation before intensifying task demands.

**Figure 8:**
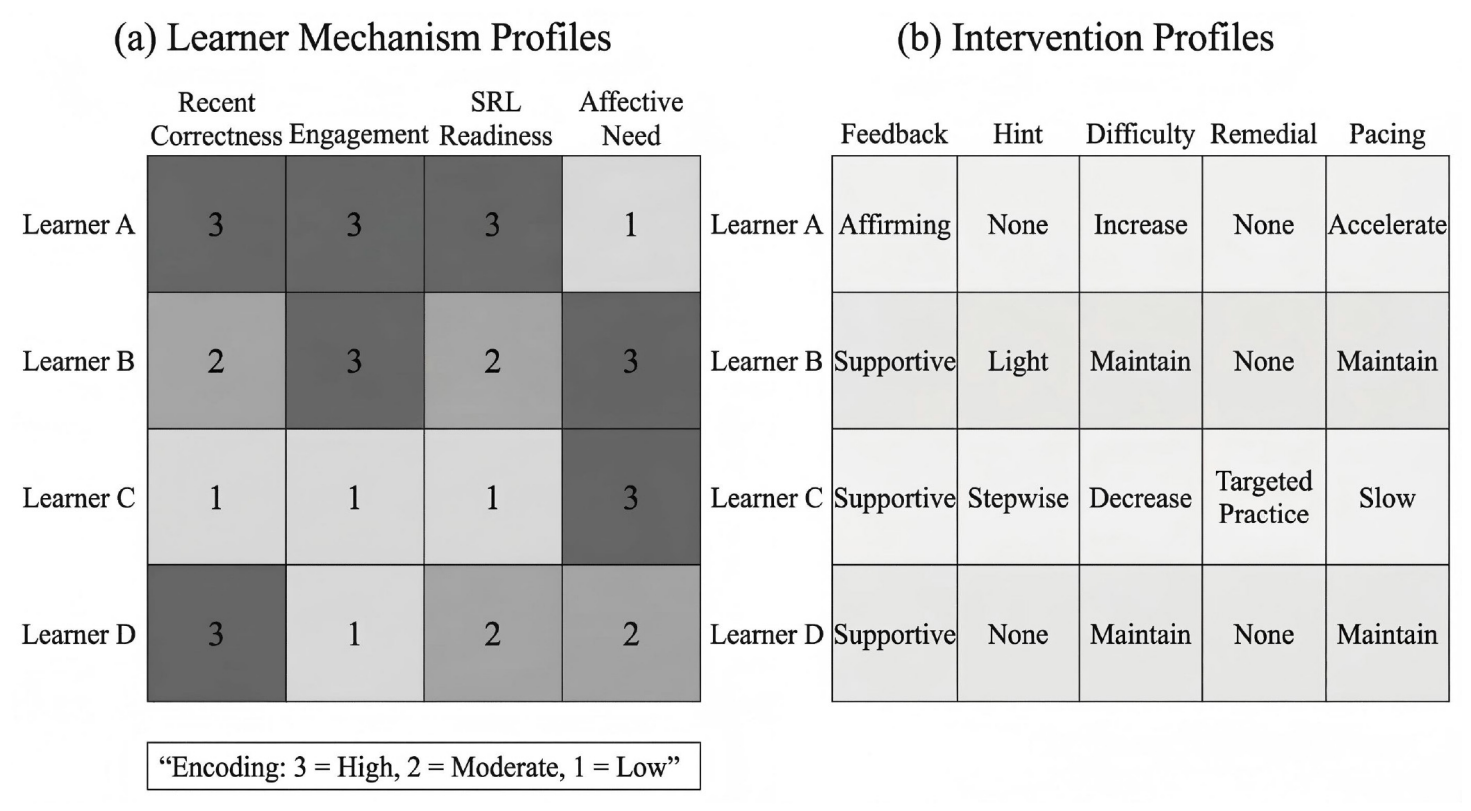
Case-level explanation analysis of MAPLe-I on representative learner profiles from the ASSISTments dataset. **(a)** visualizes the learner mechanism profiles in terms of recent correctness, engagement-related state, self-regulation-related readiness, and affective support need, where the mechanism levels are encoded as 3 = high, 2 = moderate, and 1 = low. **(b)** summarizes the corresponding intervention profiles generated by the policy head across the five pedagogical action dimensions, namely feedback selection, hint allocation, difficulty adjustment, remedial support, and pacing control. Overall, the figure shows that MAPLe-I differentiates intervention decisions not only according to observable performance, but also according to latent learner conditions, thereby providing qualitative evidence for the mechanism-aware interpretability of the proposed framework.

Figure 8 provides a more intuitive summary of the patterns reported in Table 12. Overall, these cases should be read as illustrative examples relevant to the interpretability-oriented research question of this study rather than as formal validation evidence. By linking intervention profiles not only to recent correctness but also to estimated engagement, self-regulation-related readiness, and affective support need, the framework appears to produce pedagogically differentiated responses that are consistent with its own mechanism-aware design.

### 4.8 Experimental limitations

Several experimental limitations should be acknowledged. First, the present evaluation was conducted in an offline setting rather than in a live instructional deployment. As a result, the current findings support the usefulness of the framework under offline benchmarking conditions, but do not establish real-time classroom or platform-level causal impact. Second, the three datasets differ in granularity, feature richness, and outcome structure. Although this multi-dataset design improves robustness, it also means that not all components of MAPLe-I can be evaluated identically across all datasets. Third, the proposed mechanism variables were estimated as intervention-relevant latent proxies rather than direct psychometric measurements. Therefore, the mechanism layer should be interpreted as a practically useful approximation of learner condition rather than a substitute for validated psychological assessment. Fourth, the explanation analysis is illustrative and should not be treated as formal evidence of construct validity or real-world intervention usefulness.

### 4.9 Chapter summary

In summary, the experimental results indicate that MAPLe-I consistently outperformed the selected baselines across short-term learning performance, cross-dataset generalization, long-term academic outcome prediction, intervention alignment, and potential-improvement estimation. The comparative pattern suggests that AI-driven personalized learning may be more effective when learner-state diagnosis, latent mechanism estimation, intervention policy learning, and dual-horizon outcome modeling are integrated within a unified framework rather than treated as isolated functions. These findings provide empirical evidence relevant to the research questions posed in this study and support a mechanism-aware interpretation of AI-driven personalized intervention.

## 5 Discussion

This chapter interprets the findings reported in Chapter 4 from the perspective of educational psychology and AI-driven personalized intervention. Rather than treating MAPLe-I merely as a performance-oriented computational model, the discussion focuses on what the results imply about mechanism-informed modeling under an offline benchmark design. Throughout this section, the findings are interpreted cautiously and in relation to three intertwined dimensions: the value of personalization over uniform instruction, the importance of mechanism-aware intervention over feedback-only or prediction-only approaches, and the broader implications of integrating AI methods with psychologically meaningful educational processes.

### 5.1 Main findings and their theoretical meaning

The results revealed a stable performance pattern across datasets. First, all personalized baselines outperformed the non-personalized digital intervention baseline. Second, hybrid and more advanced feedback paradigms generally outperformed simpler adaptive or generative feedback conditions. Third, learner-modeling and academic-prediction baselines such as GKTP, LASA, and XGB-SHAP achieved strong performance, but were still consistently outperformed by MAPLe-I under the present benchmark protocol. Finally, MAPLe-I showed advantages not only in short-term learning performance, but also in long-term academic outcome modeling and in the model-based Potential-Improvement Index. Taken together, these findings suggest that benchmark performance may be better supported by a coordinated framework linking learner-state diagnosis, latent proxy estimation, and adaptive support than by isolated functions such as content adaptation, feedback generation, or risk prediction alone.

From an educational psychology perspective, this pattern is relevant because it suggests that learning-relevant learner conditions may not be fully captured by observable performance history alone. At the same time, the present evidence does not establish that MAPLe-I has discovered validated psychological mechanisms or that it causally improves educational outcomes in real instructional contexts. A more appropriate interpretation is that the model's stronger benchmark performance is consistent with the value of using theory-informed learner-state proxies when structuring personalized intervention decisions.

### 5.2 Personalization as more than adaptive delivery

One of the clearest findings of the study is that the non-personalized digital intervention baseline was consistently the weakest condition across datasets. This result reinforces a longstanding educational principle: learners are heterogeneous in prior knowledge, pace, persistence, and support needs, and therefore identical instructional treatment is unlikely to be optimal for all students (Dockterman, 2018; Dumont and Ready, 2023). In the present study, even relatively simple forms of personalization outperformed the one-size-fits-all baseline, indicating that adaptation itself is educationally meaningful.

However, the results also suggest that not all personalization is equivalent. Traditional adaptive learning and feedback-centered baselines improved performance, but their gains remained smaller than those of MAPLe-I. This distinction has theoretical significance. Many digital learning systems define personalization primarily as *adaptive delivery*, that is, selecting content or progression rules based on past performance. While this approach is better than uniform instruction, it often remains behavior-reactive rather than mechanism-sensitive. In other words, such systems adapt to what the learner did, but not necessarily to *why* the learner is struggling or progressing.

MAPLe-I extends the notion of personalization in a more psychologically informed direction. In the proposed framework, personalized support is not reduced to selecting the next item or changing task difficulty. Instead, intervention is conditioned on a richer representation that includes latent proxy estimates related to engagement, self-regulation-related readiness, and affective support need. Under the present benchmark design, this pattern suggests that effective personalization in educational AI may be better understood not simply as adaptive sequencing, but as mechanism-aware pedagogical adjustment. Such an interpretation is especially relevant for educational psychology because it shifts the focus from content allocation alone to the alignment between learner condition and instructional response, while still recognizing that the learner conditions used here are computational proxies rather than validated mechanisms.

### 5.3 Why mechanism-aware intervention may matter

One of the core research questions of this study concerns whether benchmark performance is better explained when intervention-relevant learner proxies are explicitly incorporated into AI-driven personalized support. The experimental results provide evidence relevant to this question. MAPLe-I consistently outperformed feedback-only baselines, even when those baselines used adaptive logic, generative AI, or both. This pattern is consistent with the broader educational view that feedback is powerful only when it is appropriately aligned with learner condition and pedagogical purpose. It also outperformed learner-modeling and academic-prediction baselines that were strong at diagnosing knowledge states or forecasting outcomes. This pattern suggests that neither high-quality feedback generation nor accurate prediction alone is sufficient. What appears to matter in the benchmark setting is the model's ability to connect diagnosis with action through an intermediate latent-proxy layer.

Theoretically, this mechanism layer can be understood as an attempt to approximate those learner states that shape responsiveness to intervention. For example, two students may exhibit similar response accuracy while differing substantially in engagement or affective support need. A prediction model may treat them as equivalent if their observable performance is similar. A mechanism-aware intervention model, by contrast, may assign different support strategies because it recognizes that their likelihood of benefiting from challenge, scaffolding, or encouragement is different. This interpretation is highly compatible with educational psychology traditions emphasizing that instructional effectiveness depends on learner readiness, motivational state, and self-regulatory capacity rather than ability alone.

In this study, three mechanism dimensions were modeled: engagement-related state, self-regulation-related readiness, and affective support need. These constructs should not be interpreted as direct psychometric measurements, because the datasets do not provide full-scale psychological assessment. Instead, they function as latent intervention-relevant proxies inferred from learning behavior and available affect-related signals. Even so, the results indicate that incorporating such mechanism proxies improves both predictive performance and rule-alignment performance under the present evaluation protocol. This suggests that psychologically informed latent states may serve as a productive bridge between educational theory and AI-based intervention design, even when they are treated as computational approximations rather than validated constructs.

### 5.4 Educational psychological interpretation of the three mechanism dimensions

The first mechanism dimension, engagement-related state, is theoretically associated with the learner's active cognitive and behavioral involvement in the learning process. In educational psychology, engagement is commonly understood as a prerequisite for sustained learning and academic success. A learner who is behaviorally present but cognitively disengaged may not benefit fully from the same instructional materials that support an actively engaged learner. In the context of the present findings, the inclusion of engagement-related estimation likely helped MAPLe-I distinguish when learners required reinforcement, variation, or pacing adjustment to maintain productive involvement.

The second mechanism dimension, self-regulation-related readiness, captures a different but equally important educational function. Self-regulated learning involves planning, monitoring, persistence, and adaptive adjustment. Students who are not ready to sustain these processes may struggle even when content difficulty is well matched. The advantage of MAPLe-I over prediction-only models suggests that short-term correctness is not the only meaningful indicator of future improvement. Rather than demonstrating real-world intervention effectiveness, the present pattern suggests that proxy-informed modeling yields stronger predictive and rule-alignment performance than prediction-only baselines when readiness for sustained and controlled learning is represented explicitly. This interpretation is broadly consistent with educational psychology models that treat self-regulation as a proximal contributor to academic achievement.

The third mechanism dimension, affective support need, is especially relevant in digitally mediated learning environments. A student experiencing confusion, frustration, or low confidence may not need the same kind of support as a student who is cognitively challenged but affectively stable. In this sense, affective support need provides an important complement to knowledge tracing and performance prediction. The results suggest that the integration of affect-sensitive support may be one reason MAPLe-I improved Intervention Alignment Rate and the model-based Potential-Improvement Index under the present benchmark. However, this pattern should be interpreted as simulated support sensitivity rather than direct evidence that affect-aware interventions have already produced better real-world learning outcomes. Educationally, it indicates that emotional learning conditions remain relevant design variables for future AI-mediated support systems.

### 5.5 From prediction to action: a key shift in educational AI

Another important implication of the findings concerns the distinction between *prediction* and *intervention*. Many successful educational AI models are designed to estimate latent mastery, predict correctness, or identify risk. These functions are valuable, and the strong performance of GKTP, LASA, and XGB-SHAP in the experiments confirms that prediction-oriented models can provide meaningful educational information. However, the present results suggest that stronger prediction does not automatically translate into stronger real-world intervention effectiveness. In the current study, what can be shown directly is stronger benchmark prediction and stronger agreement with simulated pedagogical targets.

This distinction is critical. A model may accurately identify that a student is at risk of underachievement, but this does not ensure that the subsequent support will be pedagogically appropriate. In practical terms, an educationally useful system should connect diagnosis to support decisions; however, the present study evaluates this connection only in an offline, rule-supervised manner rather than through live intervention trials. MAPLe-I was designed to address this modeling gap by placing intervention policy learning downstream of learner-state encoding and mechanism estimation, thereby moving from descriptive intelligence toward benchmarked actionable-support modeling.

From a theoretical standpoint, this shift reflects a broader reorientation in educational AI. Rather than asking only, “Can the system predict what the learner will do?”, the more educationally meaningful question becomes, “Can the system use what it knows about the learner to provide better support?” The superior performance of MAPLe-I across short-term and long-term outcomes suggests that this intervention-oriented framing may be a more fruitful direction for future AI applications in education.

### 5.6 Short-term gains and long-term development

An especially important finding of this study is that the benefits of the proposed mechanism-aware framework were observed not only in immediate task-level performance but also in longer-horizon academic outcome modeling. This distinction is theoretically meaningful. In educational psychology, short-term correctness and long-term development are related but not identical. A learner may temporarily improve performance through intensive scaffolding or narrow task adaptation without necessarily developing stronger self-regulation, persistence, or transferable learning capacity.

The present results suggest that mechanism-aware intervention may help model this gap more explicitly in benchmark settings. When intervention is informed by engagement-related state, self-regulation-related readiness, and affective support need, instructional decisions become more sensitive to the learner's capacity to sustain progress over time rather than merely succeed on the next item. This is important because underachievement is not simply low performance; however, in the present study it is operationalized through model-based quantities rather than independently validated developmental assessments.

The Potential-Improvement Index is informative in this regard precisely because it captures predicted gain relative to baseline achievement. At the same time, it remains a model-based descriptor rather than an independently validated development-oriented indicator. Accordingly, the stronger performance of MAPLe-I on this index suggests that the framework may be useful for identifying learners whose modeled academic trajectories are especially sensitive to improved pedagogical alignment, but it does not by itself prove realized potential development.

### 5.7 Implications for AI-driven personalized learning design

The findings have several implications for the design of AI-driven personalized learning systems.

First, personalization should not be defined narrowly as content sequencing or item recommendation. Instead, future systems should incorporate richer learner representations that reflect not only performance history, but also engagement patterns, self-regulation-related signals, and affective support needs.

Second, feedback generation alone is insufficient as the sole expression of intelligence in educational AI. Even sophisticated feedback generated by adaptive or generative systems yielded weaker benchmark performance than the integrated intervention framework in the present offline setting. This suggests that future AI systems may benefit from treating feedback as one component of intervention rather than the entire intervention itself.

Third, interpretable mechanism modeling may be especially important in psychology-oriented educational research. One reason prediction models are sometimes difficult to translate into educational practice is that they provide risk estimates without clear pedagogical implications. By introducing a mechanism layer, MAPLe-I offers a structure through which computational outputs can be more meaningfully connected to educational action. Although the mechanism estimates in this study are latent and proxy-based, the framework suggests a promising direction for integrating explainability with intervention design.

Fourth, the results indicate that dual-horizon optimization is valuable. Educational support systems should ideally be evaluated not only on immediate predictive performance, but also on how well they model broader academic trajectories and how those modeled gains hold up in prospective deployments. Designing AI systems that jointly consider short-term prediction and longer-horizon academic development may therefore be a key principle for future educational technology.

### 5.8 Implications for educational psychology research

Beyond computational performance, the study also contributes conceptually to educational psychology. It suggests that AI systems can be used not only as delivery tools, but also as testbeds for mechanism-sensitive theories of learning support. If learner engagement, self-regulation-related readiness, and affective support need can be estimated from interaction data and linked to benchmark intervention outcomes, then AI-based systems may help researchers examine how instructional effects unfold dynamically rather than only through static pre-post comparisons.

This has two implications. First, it opens the possibility of studying educational mechanisms at a finer temporal granularity than is typical in traditional classroom-based research. Second, it suggests that computational models may serve as useful complements to psychological theory when they are designed around theoretically meaningful constructs rather than purely predictive targets.

At the same time, the present study does not imply that computational proxies are substitutes for rigorous psychological measurement. Instead, the findings suggest that proxy-based mechanism estimation can serve as a practical intermediate step, especially in large-scale digital environments where repeated psychometric assessment is difficult. Future research may combine interaction-based estimation with validated self-report or observational measures to strengthen construct validity.

### 5.9 Limitations

Several limitations should be acknowledged.

First, although the datasets used in this study are publicly available and suitable for modeling learning processes, they do not provide direct and comprehensive measurement of psychological constructs such as motivation, self-regulation, or emotional state. Accordingly, the mechanism variables in MAPLe-I should be interpreted as latent intervention-relevant proxies rather than validated psychological scales.

Second, the present framework was evaluated using publicly available datasets collected from different educational contexts and with different levels of granularity. Although this multi-dataset design improves robustness, it also means that not all components of the proposed framework can be assessed under exactly the same observational conditions. Future work should further validate the model using unified multimodal datasets with richer psychological and intervention annotations.

Third, the intervention policy in the present formulation is trained in an offline setting. This means that the model learns to approximate intervention decisions from historical data or constructed targets rather than being tested in a live instructional environment. While this is appropriate for a first-stage methodological study, future work should validate the framework in authentic classroom or platform-based deployment settings.

Fourth, the current manuscript does not provide a direct empirical account of deployment feasibility under real-time institutional constraints. Although the architecture is moderate in size relative to many contemporary deep models, practical use still depends on event logging quality, inference scheduling, and concurrency management in multi-user environments. This means that implementation feasibility should presently be regarded as plausible but not yet field-validated.

Fifth, although three datasets were used to improve robustness, the datasets differ in granularity and available feature types. ASSISTments is especially suitable for interaction-level and affect-related modeling, EdNet-KT1 is more appropriate for large-scale sequential validation, and OULAD emphasizes course-level outcomes. This multi-dataset design is a strength in one sense, but it also means that not all components of the proposed framework can be evaluated identically across datasets. Sixth, transferability to low-resource, classroom-only, or institution-specific settings remains uncertain. In such settings, the kinds of rich sequential traces, affect-related proxy signals, and longer-horizon outcome records used here may be unavailable or much noisier, which could materially constrain the practical usefulness of the present framework.

### 5.10 Directions for future research

Several directions for future research follow from the present findings.

First, future studies should validate MAPLe-I using real experimental implementations in online learning platforms or classroom environments. This would make it possible to examine whether the mechanism-aware intervention policy produces measurable improvements under live instructional conditions.

Second, future work should integrate richer psychological measurement. For example, validated scales of engagement, self-regulation, self-efficacy, or affect could be combined with interaction-based features to improve the construct validity of the mechanism layer.

Third, the intervention policy could be extended from supervised multi-head classification to contextual bandit or offline reinforcement learning frameworks. Such extensions may improve the model's capacity to optimize long-term educational benefit under sequential decision-making constraints.

A further priority is prospective validation in practical educational contexts. One feasible pathway would be staged deployment in learning management systems or intelligent tutoring platforms, where MAPLe-I recommendations are logged alongside teacher acceptance, student uptake, latency, and downstream outcomes. Randomized A/B studies, stepped-wedge rollouts, or teacher-in-the-loop trials would make it possible to test whether benchmark gains in prediction and rule alignment translate into measurable improvements in authentic learning environments.

Fourth, the model could be adapted for subgroup analysis, such as at-risk learners, underachieving students, or students with different levels of prior achievement. Since the concept of unlocking potential is especially relevant for learners whose current performance does not fully reflect their capacity, subgroup-based validation would be theoretically and practically valuable.

Fifth, future research may explore teacher-AI collaboration. Rather than treating AI as an autonomous intervention provider, mechanism-aware recommendations could be used to support teacher judgment, thereby combining computational precision with pedagogical expertise.

### 5.11 Chapter summary

In sum, the findings suggest that AI-driven personalized learning interventions may be modeled more effectively when they are designed not only to predict learner outcomes, but also to estimate intervention-relevant latent proxies and translate those estimates into adaptive pedagogical action. The comparative advantages of MAPLe-I across short-term performance, long-term academic outcome modeling, intervention alignment, and model-based potential-improvement estimation support a broader theoretical conclusion: academic achievement improvement in AI-supported learning environments may be better represented as a mechanism-sensitive process rather than a purely predictive one. By integrating learner-state diagnosis, latent proxy estimation, personalized intervention policy learning, and dual-horizon optimization, the proposed framework offers a conceptually richer but appropriately bounded account of how AI-supported personalized learning can be modeled in benchmark settings.

## 6 Conclusion

This study examined how AI-driven personalized learning interventions may be modeled in ways that are more transparent about learner-state representation, latent proxy estimation, intervention supervision, and longer-horizon academic outcome prediction. In response to the limitations of existing approaches that separately emphasize adaptive delivery, learner modeling, feedback generation, or academic outcome prediction, this study proposed MAPLe-I, a mechanism-aware personalized learning intervention framework that integrates learner-state diagnosis, latent proxy estimation, personalized intervention policy learning, and dual-horizon academic outcome modeling.

The study contributes to the literature in both conceptual and methodological terms. Conceptually, it reframes AI-driven personalized learning as a mechanism-sensitive intervention modeling problem rather than a narrowly predictive or feedback-centered task. Methodologically, it introduces a unified framework that translates learner-state diagnosis into mechanism-aware pedagogical action while making explicit that the learner mechanisms are computational proxies and that the intervention labels are rule-derived pseudo-labels rather than observed teacher actions.

The empirical findings reported in this study indicate that MAPLe-I achieves stronger comparative benchmark performance than the selected baselines across short-term prediction, external generalization, and long-term academic outcome modeling. At the same time, these results should be interpreted cautiously. Because the mechanism variables are proxy-based, the intervention policy is supervised with rule-derived pseudo-labels, and the evaluation is conducted offline on public datasets, the present study does not establish causal intervention effects, validated mechanism discovery, or realized improvement in students' developmental potential in authentic educational settings.

The practical implication is therefore methodological rather than triumphalist: future intelligent learning systems may benefit from making learner-condition assumptions explicit, distinguishing diagnostic modeling from intervention supervision, and evaluating short-term and longer-horizon outcomes jointly. Future work should validate these ideas with richer psychological measurement, observed intervention data, and prospective classroom or platform deployments.

In conclusion, the present study suggests that mechanism-aware personalized learning is a promising modeling direction for AI in education when interpreted with appropriate methodological restraint. By integrating learner-state diagnosis, latent proxy estimation, personalized intervention policy learning, and dual-horizon academic outcome modeling, MAPLe-I provides a clearer and more defensible benchmark framework for studying how AI-supported personalized learning may be designed and evaluated.

## Data Availability

Publicly available datasets were analyzed in this study. The ASSISTments 2012–2013 with Affect dataset is available at https://sites.google.com/site/assistmentsdata/datasets/2012-13-school-data-with-affect, with the data file accessible at https://drive.google.com/file/d/1cU6Ft4R3hLqA7G1rIGArVfelSZvc6RxY/view?usp=sharing. The EdNet-KT1 dataset is available from the official EdNet repository at https://github.com/riiid/ednet, with the KT1 download link provided at https://bit.ly/ednet_kt1. The Open University Learning Analytics Dataset (OULAD) is available from the OU Analyse repository at https://analyse.kmi.open.ac.uk/open-dataset, with the dataset download link available at https://analyse.kmi.open.ac.uk/open-dataset/download.
